# Fertility-preserving myeloablative conditioning using single-dose CD117 antibody-drug conjugate in a rhesus gene therapy model

**DOI:** 10.1038/s41467-023-41153-5

**Published:** 2023-10-12

**Authors:** Naoya Uchida, Ulana Stasula, Selami Demirci, Paula Germino-Watnick, Malikiya Hinds, Anh Le, Rebecca Chu, Alexander Berg, Xiong Liu, Ling Su, Xiaolin Wu, Allen E. Krouse, N. Seth Linde, Aylin Bonifacino, So Gun Hong, Cynthia E. Dunbar, Leanne Lanieri, Anjali Bhat, Rahul Palchaudhuri, Bindu Bennet, Megan Hoban, Kirk Bertelsen, Lisa M. Olson, Robert E. Donahue, John F. Tisdale

**Affiliations:** 1 Cellular and Molecular Therapeutics Branch, National Heart, Lung, and Blood Institute (NHLBI) / National Institute of Diabetes, and Digestive and Kidney Diseases (NIDDK), National Institutes of Health (NIH), Bethesda, Maryland, MD USA; 2 Division of Molecular and Medical Genetics, Center for Gene and Cell Therapy, The Institute of Medical Science, The University of Tokyo, Tokyo, Japan; 3 Genomics Technology Laboratory, Leidos Biomedical Research, Inc., Frederick National Laboratory for Cancer Research, Frederick, MD USA; 4 Translational Stem Cell Biology Branch, NHLBI, NIH, Bethesda, MD USA; 5 Magenta Therapeutics, Cambridge, MA USA

**Keywords:** Haematopoietic stem cells, Sickle cell disease, Preclinical research, Translational immunology

## Abstract

Hematopoietic stem cell (HSC) gene therapy has curative potential; however, its use is limited by the morbidity and mortality associated with current chemotherapy-based conditioning. Targeted conditioning using antibody-drug conjugates (ADC) holds promise for reduced toxicity in HSC gene therapy. Here we test the ability of an antibody-drug conjugate targeting CD117 (CD117-ADC) to enable engraftment in a non-human primate lentiviral gene therapy model of hemoglobinopathies. Following single-dose CD117-ADC, a >99% depletion of bone marrow CD34 + CD90 + CD45RA- cells without lymphocyte reduction is observed, which results are not inferior to multi-day myeloablative busulfan conditioning. CD117-ADC, similarly to busulfan, allows efficient engraftment, gene marking, and vector-derived fetal hemoglobin induction. Importantly, ADC treatment is associated with minimal toxicity, and CD117-ADC-conditioned animals maintain fertility. In contrast, busulfan treatment commonly causes severe toxicities and infertility in humans. Thus, the myeloablative capacity of single-dose CD117-ADC is sufficient for efficient engraftment of gene-modified HSCs while preserving fertility and reducing adverse effects related to toxicity in non-human primates. This targeted conditioning approach thus provides the proof-of-principle to improve risk-benefit ratio in a variety of HSC-based gene therapy products in humans.

## Abstract (web summary)

Successful engraftment of human hematopoietic stem cells during gene therapy requires myeloablative conditioning of the recipient, at the expense of toxicity. Authors show here that a single-dose of anti-CD117 antibody-drug conjugate achieves similar engraftment results as traditional multi-dose busulfan conditioning but preserves fertility in a non-human primate model.

## Introduction

Hematopoietic stem cell (HSC)-targeted gene therapy is potentially curative for multiple genetic diseases, including primary immunodeficiencies, hemoglobinopathies, and inherited metabolic disorders^1–4^. Among the hemoglobinopathies, sickle cell disease (SCD) is the most common single-gene disorder, caused by a point mutation in the β-globin gene (HBB:c.20 A > T), resulting in hemolytic anemia, vaso-occlusion, multi-organ damage, and early mortality^5^. While hydroxyurea and other newer drug therapies can reduce the symptoms and prevent progression to irreversible organ damage in some SCD patients via fetal hemoglobin (HbF) induction^6^ or other mechanisms, none of these correct the underlying defect, and patients require life-long administration of these medications. Thus, gene therapy to correct or replace the defective globin gene stands as an ideal approach to improve patient lives.

The desirability of a cure for SCD has resulted in the successful development of allogeneic HSC transplantation as a one-time curative treatment. However, widespread use of allogeneic transplant is limited by donor availability (only ~10% of patients with SCD in the United States have a suitable histocompatible donor available^7,8^), and multiple potentially life-threatening or debilitating complications exist with this approach, such as graft rejection or graft-versus-host disease (GVHD). Gene therapy approaches based on autologous transplantation of modified HSCs have been pursued to address these challenges. Utilizing lentiviral vectors to add an anti-sickling globin gene or a small inhibitory RNA to induce HbF production in patient’s own cells eliminates both donor dependence and risks associated with allogeneic transplantation such as GVHD^7–11^. Recently, an additional autologous HSC gene-editing therapy strategy has been successfully applied clinically via the use of engineered endonucleases to reactivate HbF production^11,12^.

Despite the broad curative potential of both allogeneic transplantation and autologous gene-addition/editing, these approaches are limited by morbidity and mortality from cytotoxic chemotherapy-based pre-transplant conditioning. In allogeneic HSC transplantation, a combination of chemotherapy and/or total body irradiation (TBI) and potent immunosuppression is required to both clear HSC niches in the marrow for efficient engraftment (myeloablation) and to prevent immunological rejection of donor cells (immunosuppression)^13^. Myeloablative doses of the alkylating agent busulfan, using a multi-day regimen, have been required to result in sufficient engraftment of gene-modified autologous CD34+ cells. Lower non-myeloablative doses have resulted in insufficient engraftment and only modest clinical impact on β-thalassemia following lentiviral gene addition^14^. Sufficient pre-treatment conditioning is essential to achieve the high levels of engraftment with gene-modified HSCs required for most clinical gene therapy applications, especially for SCD^15–17^. However, toxicity from chemotherapy-based myeloablation can result in substantial organ damage and a high risk of infertility, and can be associated with the development of secondary malignancies due to genotoxic damage to the HSCs surviving conditioning^18^.

An antibody-drug conjugate (ADC) we developed, which is designed to target HSCs with minimal collateral damage to other cells and organs, has the capacity to overcome these limitations. The exploratory CD117-ADC targets CD117 (*c-KIT*), the receptor for stem cell factor (SCF) expressed primarily on HSCs and hematopoietic progenitor cells (HPC). The antibody, with cross-reactivity to both human and non-human primate (NHP) CD117, is conjugated to a potent toxic DNA crosslinker payload from the pyrrolobenzodiazepine (PBD) class. Importantly, this ADC is engineered to enable rapid clearance, ensuring full wash-out prior to initiation of transplant.

To confirm that CD117-ADC depletes bone marrow HSCs and allows for efficient engraftment of gene-modified CD34+ cells in a NHP preclinical model with direct relevance to human HSC gene therapies, here conditioning with single-dose CD117-ADC is evaluated in an HSC gene therapy model in rhesus macaques, and results compared to myeloablative conditioning with multi-dose busulfan. These results provide the proof-of-concept for transplants using a CD117-ADC in a NHP model of gene therapy for the hemoglobinopathies and the promise of targeted conditioning with enhanced tolerability for these therapies.

## Results

### CD117-ADC targets and eliminates both human and non-human primate (NHP) CD34+ cells

**Fig. 1:**
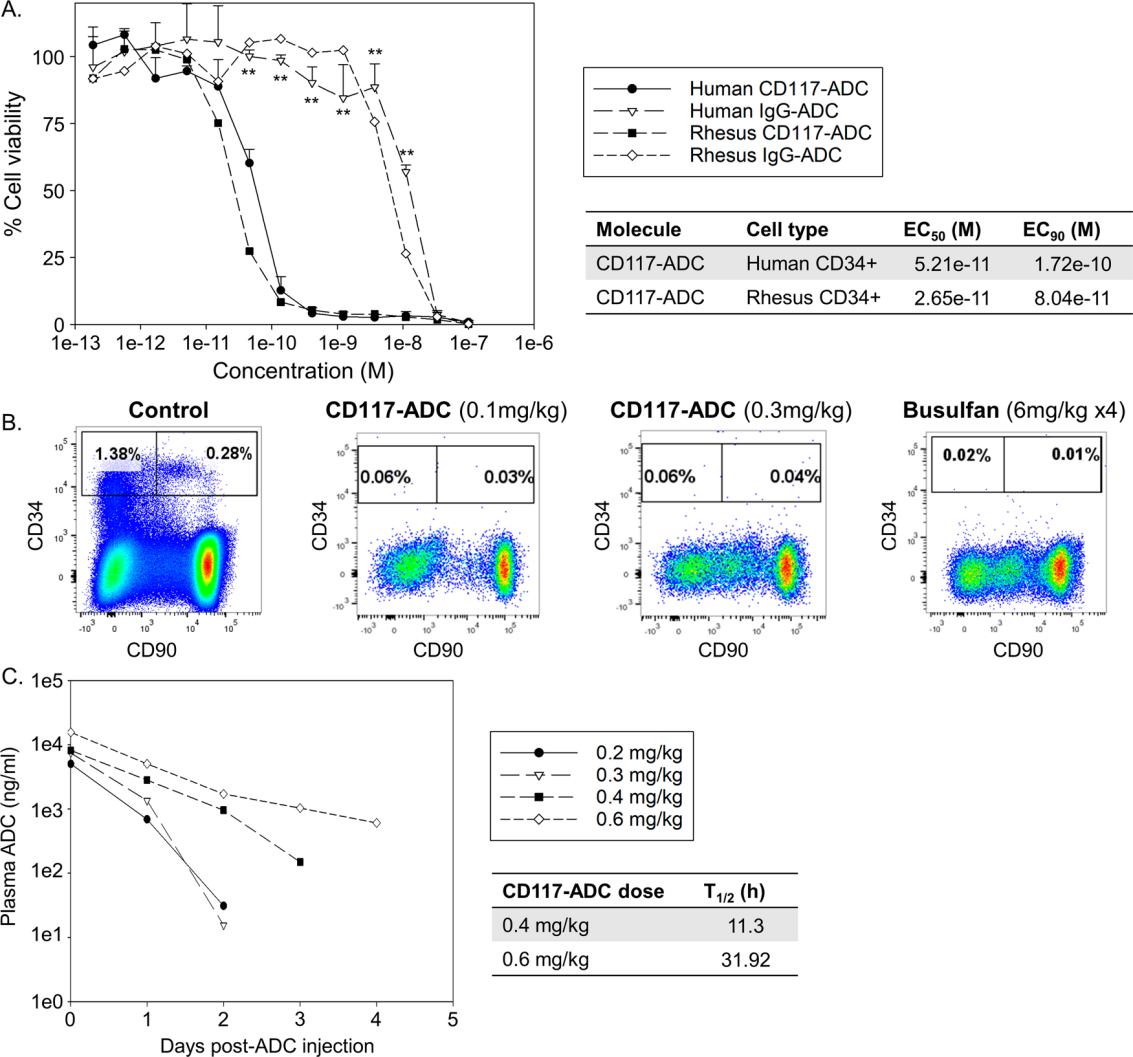
CD117-ADC depletes both human and non-human primate CD34+ cells. **A** Escalating doses of CD117-ADC were added to primary human and rhesus CD34+ cells in culture, and cell viability at day 6 was evaluated to calculate the half maximal effective concentration (EC_50_) for killing (*n* = 3 biologically independent human donors, *n* = 2 biologically independent rhesus donors). Data are presented as mean +/− standard deviation. The standard deviation was shown as error bar. **p < 0.01 evaluated by one-tailed student’s *t*-test for human CD34+ cells. *p* = 1.72e−1, 2.47e−1, 7.29x−2, 9.96e−2, 8.26e−2, 1.31e−4, 5.81e−6, 9.63e−6, 1.78e−4, 3.60e−5, 3.37e−6, 2.89e−1, and 2.98e−1, respectively (from the lower concentration). **B** Cynomolgus macaques were treated with intravenous injection of CD117-ADC (0.1 or 0.3 mg/kg × 1 day, *n* = 3 per dose) or busulfan (6 mg/kg × 4 days, *n* = 3), and bone marrow CD34 + CD90 + CD45RA- cells were quantified by flow cytometry 7 days after drug administration (*n* = 3). **C** To evaluate drug clearance, plasma concentrations of ADC in the circulation were measured after a single intravenous injection of CD117-ADC (0.2, 0.3, 0.4, and 0.6 mg/kg) in rhesus macaques (*n* = 1). The ADC concentrations for 0.2 and 0.3 mg/kg at day 3, and 0.2, 0.3, and 0.4 mg/kg at day 4 were below the limit of quantification. IgG-ADC: immunoglobulin G isotype control-conjugated ADC. Source data are provided as a Source Data file.

HSC depletion and successful transplant have previously been demonstrated by a single injection of CD117-ADC in humanized mouse models^19^. To model conditioning for human HSC gene therapies in NHPs, a CD117-ADC was selected that binds to *c-KIT* on both human and NHP cells. CD117 expression levels are similar between human and NHP CD34+ cells, analyzed by flow cytometry. This CD117-ADC potently reduced the viability of primary human and rhesus CD34+ cells following in vitro exposure, with effective concentrations 50 (EC_50_) of 52 pM and 27 pM, respectively (Fig. 1A). A single intravenous administration of CD117-ADC (0.1 and 0.3 mg/kg, *n* = 3 for each) in cynomolgus macaques was well tolerated and resulted in >99% depletion of CD34 + CD90 + CD45RA- HSCs/HPCs in bone marrow examined by flow cytometry 7 days post-administration (Fig. 1B). This degree of depletion was comparable to that seen following a standard myeloablative busulfan conditioning regimen in cynomolgus macaques (6 mg/kg, once a day for 4 consecutive days, *n* = 3). In a supplemental study where cynomolgus macaques were given a single administration of CD117-ADC at doses of 0.03, 0.1, and 0.3 mg/kg (*n* = 3 for each), CD117-ADC administration resulted in the on-target hematopoietic depletion with transient increases in aspartate aminotransferase (AST), alanine aminotransferase (ALT), lactate dehydrogenase (LDH) (1.55- to 7.7-fold) 1-day post-administration, but not other extensive toxicities associated with busulfan, including oral ulcerations, renal tubular casts, and intestinal fibrosis. No toxicity was observed in the thymus, mast cells, and skin melanocytes, which express CD117. Pharmacokinetic analysis following single-dose administration of 0.2, 0.3, 0.4, and 0.6 mg/kg CD117-ADC to rhesus macaques (*n* = 1 for each), demonstrated non-linear pharmacokinetics (Table 1) and rapid clearance from the circulation with an observed ~12-h half-life following 0.4 mg/kg administration. Based on the theoretical extrapolation of time to achieve a non-cytotoxic concentration of CD117-ADC (1.0e−3 ng/ml, the effective concentration 10), optimal timing to transplant gene-engineered engrafting CD34+ cells was predicted to be 6 or 10 days post-ADC administration of 0.2–0.3 mg/kg or 0.4 mg/kg, respectively (Fig. 1C and Table 1). By using NHP serum in killing assays, we confirmed that NHP serum did not cause cytotoxicity.

**Table 1:** Maximal concentration (Cmax) and area under the curve (AUC) of CD117-ADC in rhesus macaques

Dose (mg/kg)	Cmax (ng/ml)	AUC (ng*h/ml)
**0.2**	5038	77,347
**0.3**	7440	121,960
**0.4**	8176	190,659
**0.6**	15,620	381,895

### A single dose of CD117-ADC conditioning robustly depletes bone marrow of rhesus macaques

**Fig. 2:**
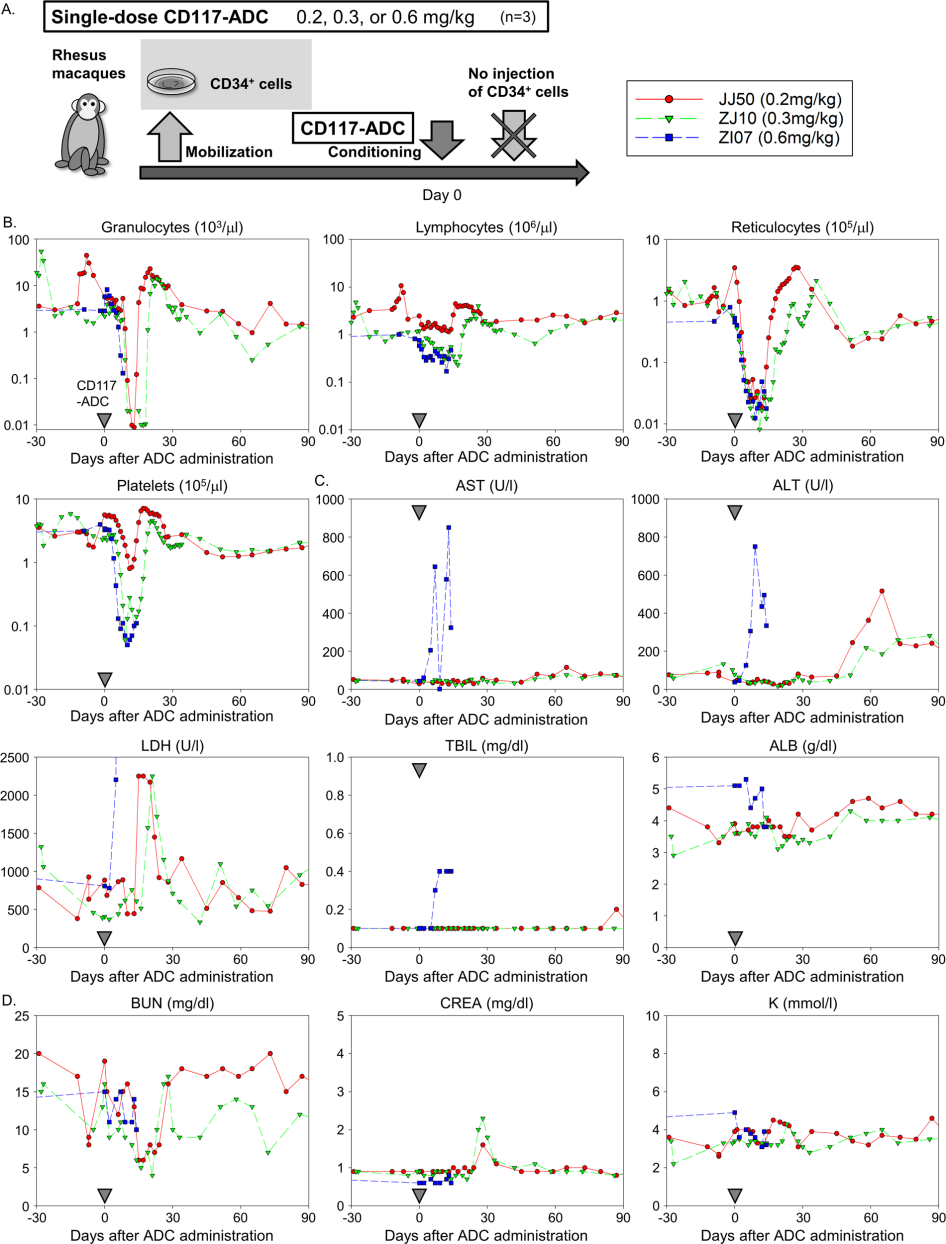
A single dose of CD117-ADC conditioning robustly depletes bone marrow of rhesus macaques. **A** Experimental design for escalating-dose CD117-ADC administration into rhesus macaques. The animals received mobilization and had CD34+ cells collected by apheresis, but these cells were not transplanted into the autologous macaques following CD117-ADC injection. **B** Peripheral blood counts in rhesus macaques before and after CD117-ADC conditioning. **C**, **D** Peripheral blood levels of liver transaminases (aspartate aminotransferase (AST) and alanine aminotransferase (ALT)), lactate dehydrogenase (LDH), total bilirubin (TBIL), albumin (ALB), blood urea nitrogen (BUN), creatinine (CREA), and potassium (K) before and after CD117-ADC administration. Source data are provided as a Source Data file.

We investigated whether escalating doses of CD117-ADC would improve HSC depletion and, thus, engraftment with gene-modified cells without increased toxicity in rhesus macaques. We administered single escalating doses of CD117-ADC (0.2, 0.3, and 0.6 mg/kg) to three rhesus macaques (JJ50, ZJ10, and ZI07, respectively) without CD34+ cell infusion and followed blood cell counts and serum chemistry parameters post-ADC injection for 90 days (Fig. 2A). In these animals, CD34+ cell mobilization was also performed before CD117-ADC injection, to account for any potential impact of the mobilization process on bone marrow ablation levels. At doses of 0.2 mg/kg and 0.3 mg/kg, transient reductions in granulocytes (<500/μ l: 5 days and 10 days, respectively), reticulocytes (<50,000/μ l: 13 days and 23 days, respectively), and platelets (<20,000/μl: none and 7 days, respectively) occurred, with no effect on lymphocyte counts (Fig. 2B and Supplementary Fig. 2B). Following a recovery period of 15 days and 19 days post-ADC administration, respectively, rebound of blood cell counts above baseline values occurred. LDH levels increased rapidly and returned to baseline, consistent with the expected damage to blood and marrow cells (Fig. 2C). There was minimal elevation of blood urea nitrogen, creatinine, and serum potassium (Fig. 2D). Initially, liver enzymes (AST and ALT) as well as other liver function tests (total bilirubin and albumin) remained normal; however, there was a minimal transient elevation of ALT in both animals around day 60 without any clinical evidence of liver dysfunction, potentially associated with the expected hepatic metabolism of components of the ADC. In contrast, ZI07 (0.6 mg/kg) suffered profound cytopenia, as well as immediate marked elevation of liver enzymes and required euthanasia on day 14 despite transfusion support. Postmortem examination showed mild enlargement of the liver with rounded edges, and diffuse centrilobular congestion and necrosis were observed in histology. Based on the reduction in targeted cell populations and positive tolerability data, doses below 0.6 mg/kg CD117-ADC were selected for conditioning prior to transplantation with lentivirally transduced CD34+ cells.

### Efficient engraftment of gene-modified CD34+ cells and robust induction of fetal hemoglobin (HbF) in rhesus macaques following CD117-ADC or busulfan conditioning

We initially assessed the impact of a single dose of 0.2 mg/kg CD117-ADC on engraftment of autologous gene-modified HSCs in two rhesus macaques (13U047 and 12U032, *n* = 2) (Supplementary Figs. 1, 2A, and 3A). Both animals achieved hypocellular bone marrow on the day of transplant, with reduction in the counts of granulocytes, reticulocytes, and platelets, but observed only modest levels of gene-marked cells in the periphery (vector copy number [VCN] 0.05 ± 0.01).

**Fig. 3:**
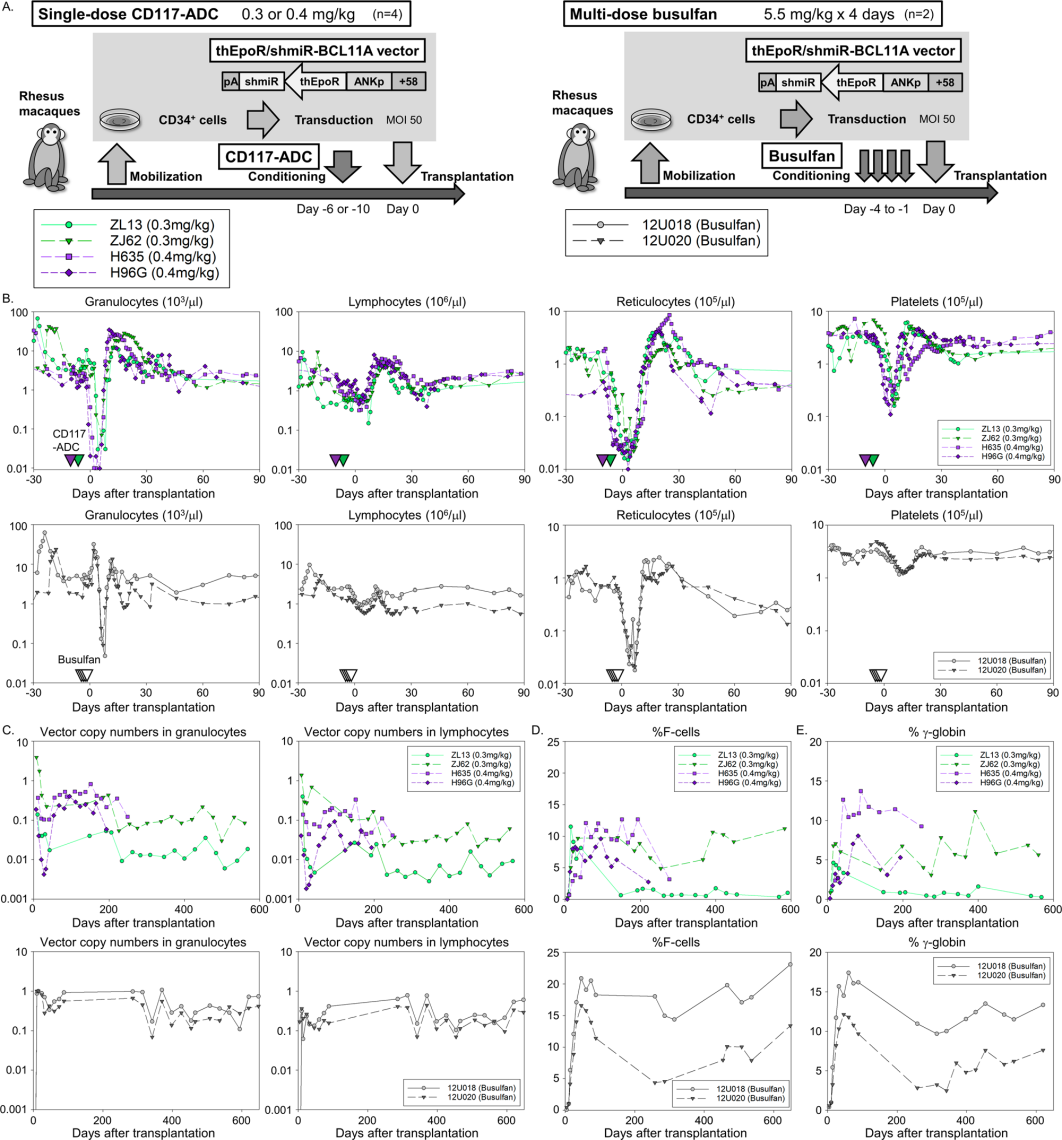
Efficient gene marking and robust HbF induction after CD117-ADC conditioning in a rhesus gene therapy model compared to busulfan. **A** Schema of autologous CD34+ cell transplantation with thEpoR and shmiR-BCL11A lentiviral vector transduction (MOI 50). Transplantation occurred either 1 day after myeloablative busulfan conditioning (5.5 mg/kg × 4 days) in rhesus macaques (*n* = 2), 12U018 and 12U020, or 6 or 10 days after a single injection of 0.3 or 0.4 mg/kg CD117-ADC in rhesus macaques (*n* = 4) (0.3 mg/kg in ZL13 and ZJ62, 0.4 mg/kg in H635 and H96G), respectively. **B** Blood counts in rhesus macaques before and after transplantation. **C** Vector copy number (VCN) in granulocytes and lymphocytes post-transplantation, evaluated by quantitative polymerase chain reaction (qPCR). **D** HbF-positive red blood cells (F-cells) post-transplantation, evaluated by flow cytometry. **E** HbF (γ-globin) amounts at the protein level in red blood cells post-transplantation, evaluated by reversed-phase high-performance liquid chromatography (HPLC). pA: a polyadenylation signal, ANKp: an erythroid-specific ankyrin-1 promoter, +51: an erythroid-specific enhancer in the *BCL11A* gene. Source data are provided as a Source Data file.

We therefore evaluated higher doses of single-dose CD117-ADC in four macaques (0.3 mg/kg for ZL13 and ZJ62, and 0.4 mg/kg for H635 and H96G, *n* = 4) as conditioning agent for autologous lentivirally transduced CD34+ cells and compared the results directly to busulfan conditioning (5.5 mg/kg × 4 days for 12U018 and 12U020, *n* = 2) (Fig. 3A). Similar amounts of mobilized rhesus CD34+ cells (CD117-ADC 6.4 ± 2.9e6/kg vs. busulfan 4.1±0.1e6/kg, n.s.) were transduced with a lentiviral vector encoding a short hairpin RNA embedded in a microRNA targeting the B-cell lymphoma/leukemia 11A (*BCL11A*) gene (shmiR-BCL11A) and a truncated human erythropoietin receptor (thEpoR), which we have previously reported to result in stable HbF induction following myeloablative TBI conditioning in our rhesus macaque model^20^. The transduced cells, with VCN assessed in vitro as 10.1 ± 3.8 for the cells to be transplanted into animals following ADC-CD117 conditioning and 10.2 ± 7.3 for the cells to be transplanted following busulfan conditioning were infused into the autologous animals 6 or 10 days after ADC conditioning (0.3 or 0.4 mg/kg, respectively) or 1 day after busulfan conditioning^15,16^. The time course of blood cell count suppression and recovery was similar in these CD117-ADC animals and the busulfan-conditioned animals (Fig. 3B, and Supplementary Fig. 2C, D), except that a marked reduction of platelet counts was observed only with CD117-ADC conditioning. A rebound of blood cell counts above normal was observed again in all animals after CD117-ADC conditioning, but not after busulfan conditioning. At 1 month post-transplant, T cells were dominant for most animals with either CD117-ADC or busulfan conditioning as compared to B cells, and the B and T cell balance was recovered 3–6 months post-transplant (Supplementary Fig. 4). Two months post-transplant, substantial levels of vector-containing granulocytes were observed long-term in one of two animals receiving 0.3 mg/kg CD117-ADC (ZJ62, VCN 0.16) and in both animals receiving 0.4 mg/kg (H635, VCN 0.46 and H96G, VCN 0.21), in a similar range to that observed in the two animals receiving busulfan conditioning (0.44 ± 0.17, n.s.) (Fig. 3C). Robust HbF induction was also detected in the three CD117-ADC-conditioned animals with substantial VCN as measured by both HbF-positive cells, comparable to the busulfan conditioned animals (F-cells 8.5 ± 1.8% vs. 13.7 ± 5.8%, n.s.) (Fig. 3D), and high-performance liquid chromatography (HPLC) measurement of total HbF in CD117-ADC vs. busulfan conditioned animals (8.0 ± 2.9% vs. 11.1 ± 5.2%, n.s.) (Fig. 3E). In ZL13 (0.3 mg/kg CD117-ADC), much lower gene marking (VCN in granulocytes 0.02) was observed (Fig. 3C), along with weak HbF induction (F-cells 1.0% and HbF amounts 0.9%) (Figs. 3D, E). There was the positive correlation between HPLC-measured HbF levels and granulocyte VCN of the thEpoR/shmiR-BCL11A vector in the rhesus model (*p* < 0.01) (Supplementary Fig. 5).

Importantly, unlike busulfan conditioning, CD117-ADC conditioning resulted in minimal toxicity (Supplementary Fig. 3B, C). Increased levels of LDH were observed at both the 0.3 and 0.4 mg/kg doses 10 days after transplant which resolved by 30 days post-transplant. High diversity of gene-modified cells (Simpson’s diversity index >0.99) without any large clone (maximal integration site 0.3–3.7%) was observed by integration site analysis 1 year after transplantation with both CD117-ADC (0.3–0.4 mg/kg) and busulfan conditioning (Supplementary Fig. 6). Erythropoietin (Epo) levels were not elevated from the use of the thEpoR/shmiR-BCL11A vector 14–22 months post-transplant with CD117-ADC and busulfan conditioning, compared to pre-transplant (Supplementary Fig. 7). These data demonstrate that 0.3 mg/kg CD117-ADC is a minimum effective dose to facilitate robust engraftment, and 0.4 mg/kg CD117-ADC appears more reliable at ensuring sufficient engraftment of gene-modified cells with only minimal toxicity.

### Fertility in animals following CD117-ADC conditioning

**Fig. 4:**
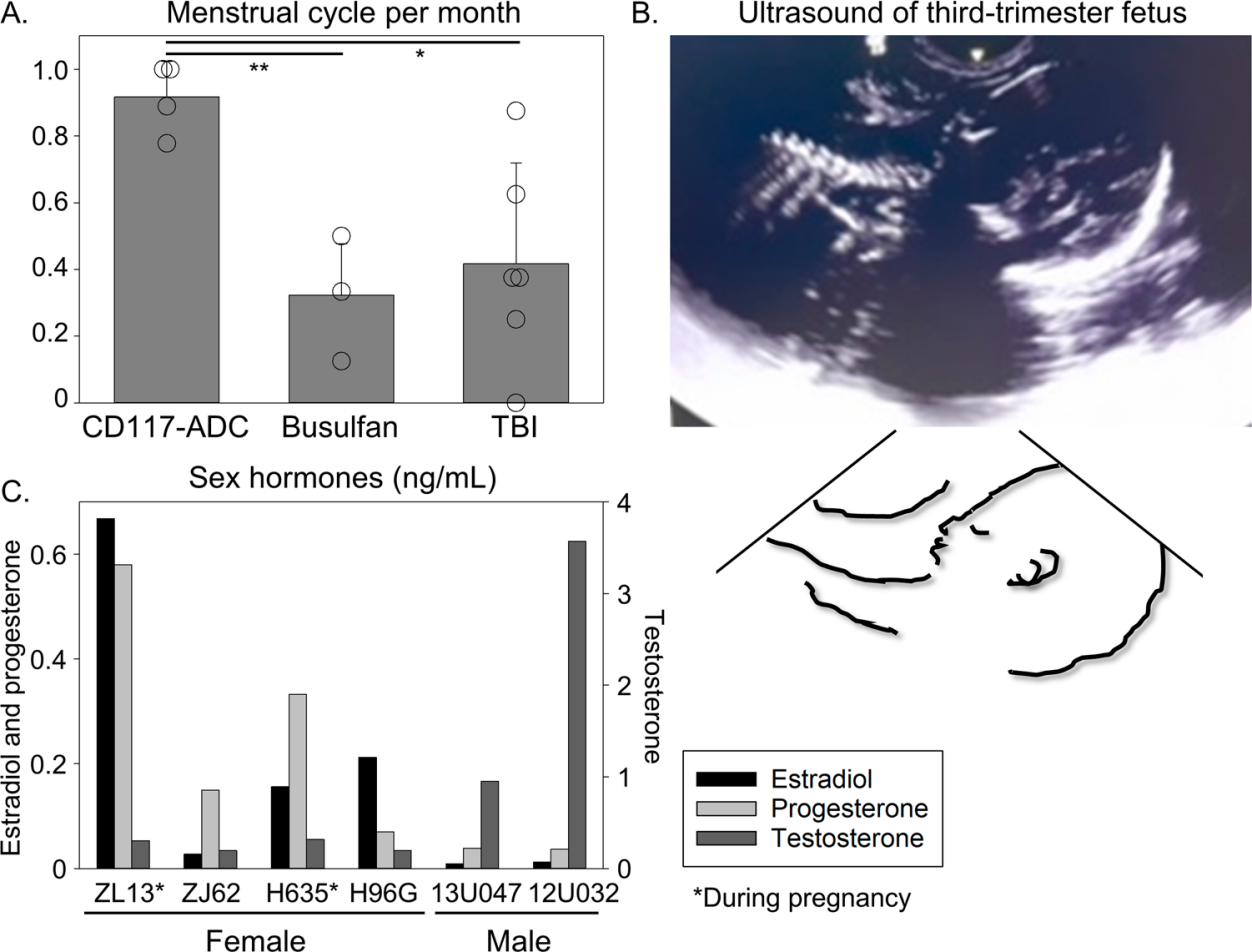
Pregnancy in transplanted macaques following CD117-ADC conditioning. **A** The menstrual cycle per month after rhesus HSC transplantation with 0.3–0.4 mg/kg CD117-ADC (*n* = 4 biologically independent animals), myeloablative busulfan (5.5 mg/kg × 4 days, *n* = 4 biologically independent animals), or myeloablative total body irradiation (TBI, 4.5–5.0 Gy × 2 days, *n* = 6 biologically independent animals) conditioning. Data are presented as mean +/− standard deviation. The variances of menstrual cycle per month are equivalent among three groups. **p < 0.01, *p < 0.05 evaluated by Tukey’s HSD test (two-tailed). *p* = 8.84e−3 between CD117-ADC and Busulfan, *p* = 1.44e−2 between CD117-ADC and TBI, and *p* = 8.01e−1 between Busulfan and TBI. **B** Ultrasound image and schema of the fetus in a pregnant macaque (ZL13). **C** The sex hormones (estradiol, progesterone, and testosterone) in rhesus serum after mating following transplantation with CD117-ADC conditioning. ZL13 and H635 were pregnant. Source data are provided as a Source Data file.

Infertility is one of the major complications from conventional myeloablative conditioning that limits uptake of potentially curative therapies; therefore, we evaluated fertility in transplanted animals with CD117-ADC. When monitored daily for signs of menstrual bleeding for consecutive 8 ± 2 months, the menstrual cycles of all four female macaques (ZL13, ZJ62, H635, and H96G) were maintained post CD117-ADC conditioning in contrast to available data from historical TBI- and busulfan-conditioned animals from our group (Fig. 4A). To confirm fertility, all transplanted animals post CD117-ADC including females and males (13U047 and 12U032) were housed for mating among a rhesus breeding colony. Two female macaques were in their third trimester of pregnancy (ZL13 and H635), confirmed by ultrasound (Fig. 4B) and the two male animals were paired with pregnant females at the first check of the breeding season. Both estradiol and progesterone were elevated in the pregnant animals (Fig. 4C). Finally, we confirmed that two ADC females (ZL13 and H635) gave birth. Importantly, an ADC female (H635) was paired with an ADC male (13U047). These data demonstrate that CD117-ADC at sufficient doses for the engraftment of HSCs can maintain fertility for both genders in a NHP model.

## Discussion

We have developed an anti-CD117 antibody-drug conjugate that demonstrates potent activity in depleting both human and rhesus macaque HSCs/HPCs. The optimized CD117-ADC drug was engineered for rapid clearance, and a single dose was shown to be potently effective in depleting HSCs/HPCs, allowing efficient engraftment following transplantation of genetically modified autologous CD34+ cells. The CD117-ADC had a favorable safety profile for autologous HSC transplantation conditioning, including sparing of peripheral lymphocytes and preservation of fertility in rhesus macaques. In our rhesus model of autologous HSC gene therapy, a single dose of CD117-ADC enabled long-term stable engraftment of gene-modified CD34+ cells, achieving similar levels of genetically modified cells as compared to conditioning with myeloablative doses of busulfan. Robust HbF induction at the protein level was also confirmed following CD117-ADC conditioning. These proof-of-concept studies validate the use of a CD117-ADC approach for targeted HSC/HPC depletion prior to transplantation and support its use as a new conditioning agent for autologous HSC gene therapies. CD117-ADC offers a potentially safer conditioning approach avoiding the short- and long-term complications of classic, non-specific conditioning regimens with an improved risk-benefit profile, thus enabling more patients to benefit from these potentially curative therapies.

We observed that CD117-ADC conditioning transiently suppresses granulocytes, reticulocytes, and platelets but not lymphocytes in rhesus macaques, demonstrating that CD117-ADC is myeloablative but not immunoablative. Reductions of blood counts were observed in both rhesus and cynomolgus macaques; however, there were variabilities of engraftment efficiency among monkeys that were similar to our historical data. Importantly, the observed time-course of recovery of these populations is consistent with the characterization of CD117-ADC demonstrating targeted depletion with rapid clearance, key features for a conditioning agent. As myeloablation is sufficient for engraftment of autologous CD34+ cells^15–17^, CD117-ADC conditioning should be suitable for both autologous HSC gene-addition and gene-editing therapies. In contrast, both myeloablation and immunosuppression are required for allogenic transplantation to ensure engraftment of donor HSCs and prevent immune-mediated rejection. As a result, an immunosuppressive agent would likely be required in addition to CD117-ADC conditioning in allogeneic HSC transplantation, or targeting of a pan-leukocyte antigen such as CD45 with an ADC, as has been explored in murine models^21^. For immunodeficient patients receiving allogeneic HSC transplants such as those with severe combined immunodeficiencies (ClinicalTrials.gov: NCT02963064), CD117-ADC conditioning alone might be sufficient.

In this study, after blood cell counts recovered, there was a transient increase to levels higher than the baseline for granulocytes, reticulocytes, and platelets, all cell lineages dependent on continuous production from HSCs/HPCs, suggesting an overshoot in recovery following engraftment in the context of CD117-ADC conditioning. Interestingly, even lymphocytes in the blood increased over baseline following recovery from CD117-ADC conditioning, despite no initial reduction in circulating lymphocytes following CD117-ADC, and no predicted impact on T-cell homeostasis at least in the short-term. This rebound in blood cell counts has not been observed in our experience with hundreds of autologous transplants in rhesus macaques following busulfan or TBI conditioning^16, 22,23^. At the timing of lymphocyte rebound, T cells were dominant for most animals, compared to B cells; however, T-cell dominance was also observed in busulfan-conditioning animals without lymphocyte rebound. A comprehensive understanding of the change in cytokine levels following CD117-ADC conditioning may help to explain the transient increase of all circulating blood cell lineages. This transient increase may be a result of enhanced preservation of hematopoietic niches by a targeted conditioning approach versus conventional non-targeted chemotherapy conditioning^24^, such that upon clearance of the ADC, these populations sufficiently recover by virtue of their limited toxic exposure.

We observed the positive correlation between HbF induction levels in HPLC analysis and granulocyte VCN of the thEpoR/shmiR-BCL11A vector in the rhesus model (*p* < 0.01). The ~1.1 VCN in granulocytes allowed for 20% HbF induction in rhesus red blood cells, which is thought to be a therapeutic level in SCD. A therapeutic level of 1-2 VCN was also observed in our SCD gene therapy trial with the β^T87Q^-globin vector^2^. However, in contrast to results with lentiviral vectors in humans in our gene therapy trial, >10-fold higher VCN in infusion products of transduced CD34+ cells is required to achieve anticipated VCN in vivo in our rhesus transplantation model^25^. The NHPs are a preclinical model, demonstrating that CD117-ADC results in hematopoietic depletion similar to busulfan, along with minimal toxicity less than busulfan.

Recently, two patients with SCD developed myelodysplastic syndrome (MDS)/acute myeloid leukemia (AML) several years after lentiviral HSC gene therapy with myeloablative busulfan conditioning. The exhaustive studies did not implicate vector insertion as driving leukemic transformation in these patients^9,10^. Pre-leukemic clones that are likely generated by the SCD disease process could be selectively expanded or further damaged with cyto-genotoxic conditioning, increasing the risk of MDS/AML development^18,26,27^. Theoretically, CD117-ADC conditioning (in particular, ADC with a cytotoxic payload without direct DNA targeting, for example MGTA-117 which utilizes an amanitin-based payload and was tested in a Phase 1/2) should be less likely to increase the risk of this complication, given its lack of direct DNA damage in the process of targeting HSCs/HPCs. In addition, CD117 is expressed by various malignant cells, including leukemic cells^28^. Therefore, selectively targeting it could possibly deplete endogenous pre-leukemic clones during conditioning, which is especially important considering that measurable residual disease (MRD) following treatment is associated with higher risk of relapse and shorter survival^29^.

In January 2023, Magenta voluntarily paused dosing in its MGTA-117 Phase 1/2 clinical trial in patients with relapsed/refractory AML and MDS, after the last participant dosed in Cohort 3 experienced a Grade 5 serious adverse event (respiratory failure and cardiac arrest resulting in death), deemed to be possibly related to MGTA-117. In February 2023, Magenta discontinued the MGTA-117 Phase 1/2 clinical trial in patients with AML and MDS. It is important to note that MGTA-117 utilized an amanitin payload which is different from the PBD payload used in the NHP conditioning studies outlined in this publication.

CD117-ADC at doses resulting in robust engraftment with genetically modified cells is also not associated in our studies or predicted to cause infertility, veno-occlusive disease, pulmonary dysfunction, mucositis, or seizures, in contrast to busulfan. Generally, side effects from TBI or busulfan-based conditioning at myeloablative doses result in significant morbidity and require prolonged inpatient-level medical care in both rhesus macaques and humans due to toxicity to organs and tissues that are dependent on proliferating cells, such as the gut, or that are directly damaged via other pathways linked to busulfan or TBI, i.e., liver and kidneys. The rhesus macaques treated with CD117-ADC at doses of 0.4 mg/kg or less maintained normal appetite and activity even during suppression of blood counts after CD117-ADC conditioning.

However, on-target but undesired effects of CD117-ADC are possible due to CD117 expression by a limited number of other cell types, including mast cells, melanocytes, and germ cells^28^. Previously, mast cell degranulation was observed clinically as part of a hypersensitivity reaction following administration of LOP628, a preliminary type of CD117-ADC^30^. In this study, our CD117-ADC was specifically designed to avoid mast cell degranulation, and impacts of anti-CD117 mast cell degranulation were not observed in preclinical NHP models. The fragment crystallizable (Fc) region in the heavy chain was mutated to abolish neonatal Fc receptor binding and silence the effector function. The engineered antibody allowed for no degranulation of mast cells in vitro. In fact, no toxicity was observed at mast cells as well as thymus and skin melanocytes in cynomolgus macaques following a single administration of CD117-ADC (0.03, 0.1, and 0.3 mg/kg). These data suggest that a single dose of CD117-ADC for conditioning is not associated with any long-term toxicity to mast cells and melanocytes, although further evaluation is needed. In addition, the undesired effects on germ cells might induce infertility, similar to TBI and/or chemotherapy-based conditioning. In our rhesus model post-ADC conditioning, the ovaries appeared normal at necropsy (ZJ10 in 0.3 mg/kg CD117-ADC and ZI07 in 0.6 mg/kg CD117-ADC), and all four female recipients continued menstruating after CD117-ADC conditioning, in contrast to the busulfan-conditioned recipients where amenorrhea is generally observed in humans^31^ and rhesus. After transfer to a rhesus breeding colony, two of four female macaques (ZL13 and H635) gave birth, and both males were paired with pregnant females (one with H635 giving birth). These data demonstrate that CD117-ADC conditioning can allow for not only the engraftment of HSCs but also the maintenance of fertility, even when both the male and female underwent conditioning and transplantation. Fertility preservation should expand the option of HSC gene therapy, since infertility is a major reason why young patients do not select it.

In summary, we demonstrated that a single dose of CD117-ADC allows for efficient engraftment of gene-modified CD34+ HSCs in a rhesus gene therapy model, achieving similar levels as the current clinical standard, myeloablative busulfan conditioning regimen. Robust HbF induction was also confirmed at the protein level in our rhesus gene therapy model with CD117-ADC conditioning. The transplanted animals with CD117-ADC conditioning maintained their fertility. These results suggest that an ADC-based targeted approach for safer conditioning could improve the risk-benefit profile in HSC gene therapy.

## Methods

### Antibody-drug-conjugate (ADC)

The variable regions in heavy and light chains (VH/VL) of an anti-human CD117 antibody (cross-reactive to cynomolgus macaque and rhesus macaque CD117) were grafted onto a human immunoglobulin G1 (IgG1) Fc region mutated in the heavy chain to abolish neonatal Fc receptor (FcRn) binding to enhance in vivo clearance^32^. Cysteine residues^33^ incorporated in the Fc region for site-specific conjugation to the PBD linker payload tesirine^34^ yielded CD117-ADC (Magenta Therapeutics, Cambridge, MA, USA). An anti-hen egg lysozyme isotype control with matching engineering and conjugation was used to generate Isotype-ADC which served as a control (Magenta Therapeutics).

### Human and non-human primate CD34+ cell killing assay

Primary human CD34+ cells were purchased from STEMCELL technologies (Vancouver, BC, Canada). The cells were obtained using Institutional Review Board (IRB)-approved consent forms and protocols. The steady-state human bone marrow CD34+ cells (3 donors, STEMCELL technologies) or purified mobilized CD34+ cells from two healthy rhesus macaques were cultured (2500 cells/well in 384-well plate) in serum-free StemSpan SFEM media (45 μl, STEMCELL Technologies) supplemented with 100 ng/ml each of Interleukin 6 (IL-6), fms-related tyrosine kinase 3 ligand (FLT3L), and thrombopoietin (TPO). Escalating doses of CD117-ADC as well as IgG isotype control-conjugated ADC were added (5 μl ADC added to 45 μl cells). After 6 days of culture, the number of viable CD34 + CD90+ cells were evaluated by flow cytometry using 7-Aminoactinomycin D (7-AAD as a viability marker, Biolegend, San Diego, CA, USA), CD34 BV785 (clone 561, BioLegend), and CD90 APC (Clone 5E10, BioLegend) detection antibodies. Cells were acquired on a BD Celesta Instrument and analyzed with FloJo. The EC_50_ and EC_90_ values were calculated with GraphPad Prism software.

### Lentiviral vector design and preparation

For efficient transduction in both human and rhesus CD34+ cells, chimeric human immunodeficiency virus type 1-based lentiviral vectors (χHIV vectors) were prepared in 293 T cells (American Type Culture Collection (ATCC), Manassas, VA, USA) with plasmid transfection and concentrated by ultracentrifugation^22,23^. In the β-globin vector, the human β-globin gene is expressed by the β-globin promoter with locus control regions^35,36^. In the thEpoR/shmiR-BCL11A vector, a chimeric sequence between thEpoR and shmiR-BCL11A is driven by the erythroid-specific ankyrin-1 promoter with an erythroid-specific BCL11A enhancer, allowing for BCL11A interference and stable HbF induction^20^. The lentiviral titers were evaluated in transduced HeLa cells (ATCC) with the SIN-LTR probe/primers by quantitative polymerase chain reaction (qPCR, QuantStudio 6 Flex Real-Time PCR System, Thermo Fisher Scientific, Waltham, MA, USA)^25^.

### Depletion of CD34 + CD90 + CD45RA- cells in cynomolgus macaque

Cynomolgus macaques^37^ were treated with intravenous injection of CD117-ADC (0.1 or 0.3 mg/kg × 1 day) or busulfan (6 mg/kg × 4 days), and bone marrow CD34 + CD90 + CD45RA- cells were quantified by using antibodies against CD34 BV785 (clone 561, BioLegend, 1:300), CD90 APC (clone 5E10, BioLegend, 1:300), and CD45RA VioBlue (clone T6D11, Miltenyi Biotec, 1:50) in flow cytometry 7 days after drug administration (n = 3 per group) and compared to historical phosphate-buffered saline (PBS)-treated naïve animals. Bone marrow cellularity was assessed by complete blood count analysis to determine white blood cells per ml of marrow. Flow cytometry was used to determine the frequency of CD34 + CD90 + CD45RA- cells of total CD45+ cells. The absolute number of CD34 + CD90 + CD45RA- was determined by multiplying the frequency of CD34 + CD90 + CD45RA- cells (out of total CD45 cells) with the bone marrow cellularity in cells per ml.

### Autologous CD34+ cell transplantation with lentiviral transduction in a non-human primate

We had developed an autologous CD34+ cell transplantation model with lentiviral transduction in rhesus macaques^22,23^, following the guidelines set out by the Public Health Service Policy on Humane Care and Use of Laboratory Animals under a protocol (H-0136) approved by the Animal Care and Use Committee of National Heart, Lung, and Blood Institute (NHLBI). Rhesus CD34+ cells were mobilized with granulocyte-colony stimulating factor (G-CSF, 20 μg/kg for 5 days, Amgen, Thousand Oaks, CA, USA) and plerixafor (1 mg/kg on day 5 of G-CSF, Amgen) and isolated by magnetic separation. The mobilized rhesus CD34+ cells (4e6 cells/ml) were pre-stimulated in X-VIVO10 culture media (Lonza, Basel, Switzerland) including 100 ng/ml each of SCF (R&D Systems, Minneapolis, MN, USA), FLT3L (R&D Systems), and TPO (R&D Systems) for 1 day and transduced with lentiviral vectors encoding either human β-globin gene (13U047 and 12U032)^35^ or thEpoR/shmiR-BCL11A cDNA (12U018, 12U020, ZL13, ZJ62, H635, and H96G)^20^ at a multiplicity of infection (MOI) 50 in the fresh culture media supplemented with the same cytokines as well as 10 μ M prostaglandin E2 (R&D Systems) and 100 μ g/ml poloxamer 407 (Sigma-Aldrich, Saint Louis, MO, USA)^35^. The next day, transduced rhesus CD34+ cells were cryopreserved by Recovery Cell Culture Freezing Media (Thermo Fisher Scientific). After conditioning with either a single intravenous injection of CD117-ADC (0.2 mg/kg in 13U047 and 12U032, 0.3 mg/kg in ZL13 and ZJ62, and 0.4 mg/kg in H635 and H96G) or four-dose intravenous injections of busulfan (5.5 mg/kg × 4 days in 12U018 and 12U020), the frozen CD34+ cells with lentiviral transduction were intravenously infused into autologous macaques (6 days post-ADC at 0.2-0.3 mg/kg, 10 days post-ADC at 0.4 mg/kg, and 1 day post-busulfan). In addition, following G-CSF and plerixafor-based mobilization and the harvest of CD34+ cells, CD117-ADC was intravenously injected into rhesus macaques (0.2 mg/kg in JJ50, 0.3 mg/kg in ZJ10, and 0.6 mg/kg in ZI07) without autologous CD34+ cell transplantation. The plasma concentrations of ADC were measured by enzyme-linked immunosorbent assay (ELISA) using a human therapeutic IgG1 ELISA kit (Cayman Chemical, Ann Arbor, MI, USA) following a single CD117-ADC administration at 0.2 mg/kg (JJ50), 0.3 mg/kg (ZJ10), 0.4 mg/kg (H635), and 0.6 mg/kg (ZI07). Pharmacokinetic parameters were estimated with Phoenix pharmacokinetic software (Certara, Princeton, NJ, USA) using a non-compartmental approach consistent with the intravenous route of administration. Half-life (T½) in hours, maximal concentration (Cmax) in ng/ml, area under the curve (AUC) in ng*hr/ml, clearance (Cl_obs) in ml/hr/kg, and volume of distribution (Vz_obs) in ml/kg parameters were calculated.

During transplantation, complete blood counts (white blood cells, granulocytes, lymphocytes, red blood cells, hemoglobin concentrations, hematocrit, reticulocytes, and platelets) and biochemistry tests (AST, ALT, LDH, total bilirubin (TBIL), albumin (ALB), blood urea nitrogen (BUN), creatinine (CREA), and potassium (K)) were evaluated in peripheral blood samples. Based on blood counts, transplanted animals were maintained by whole blood transfusion and platelet-rich plasma transfusion. Aspiration samples from bone marrow were evaluated by hematoxylin and eosin staining. After engraftment of gene-modified cells, VCN was evaluated in granulocytes and lymphocytes with the SIN-LTR probe/primers by qPCR^25^. The percentage of F-cells (clone 2D12, BD Biosciences, 1:30) was evaluated by flow cytometry (FACSCanto, BD Biosciences) (Supplementary Fig. 8A)^20^. Live subsets of peripheral blood mononuclear cells were gated using 7-AAD (BD Biosciences, 1:20) staining and analyzed by using antibodies against CD4 PE (clone L200, BD Biosciences, 1:20), CD8 BUV737 (clone G42–8, BD Biosciences, 1:20), CD11b BUV395 (clone D12, BD Biosciences, 1:40), CD14 FITC (clone M5E2, BD Biosciences, 1:10), CD16 PE-Cy7 (clone 3G8, BD Biosciences, 1:40) CD18 R718 (clone 6.7, BD Biosciences, 1:40), CD20 APC-H7 (clone 2H7, BD Biosciences, 1:20), CD45 V450 (clone D058-1283, BD Biosciences, 1:40), and CD56 APC (clone B159, BD Biosciences, 1:10) (Supplementary Fig. 8B). The amounts of γ-globin production in rhesus red blood cells were evaluated by reversed-phase HPLC^38,39^. Serum Epo levels were evaluated before and after transplantation (14–22 months post-transplant) by human Epo ELISA kit (STEMCELL Technologies). The Epo levels were multiplied by 2.5, since the sensitivity of rhesus Epo is ~40% in human Epo ELISA^40^.

Mating was performed among the colony at the Alpha Genesis Primate Research Center (Yemassee, SC, USA). Estradiol, progesterone, and androstenedione were analyzed at the Wisconsin National Primate Research Center (Madison, WI, USA).

The animals are specific pathogen-free. They are individually housed while in the intensive care unit (ICU) during transplantation, pair-housed upon recovery, and group-housed for breeding. When euthanasia is required, the animal is sedated with ketamine and/or telazol, and receives an overdose of pentobarbital (80 mg/kg IV or IC), followed by potassium chloride (100 mg/kg IV or IC).

### Integration site analysis

Integration site analysis was performed as previously described^41^. In brief, DNA was extracted from the granulocyte fraction of peripheral blood 1 year post-transplantation with CD117-ADC conditioning (0.3 mg/kg in ZL13 and ZJ62, 0.4 mg/kg in H635 and H96G) and myeloablative busulfan conditioning (12U018 and 12U020). The DNA sequences around lentiviral integration sites were linearly amplified and analyzed by next-generation sequencing. Integration sites were mapped to the rhesus genome assembly rheMac10. Due to limited annotation in rheMac10, human gene names and RefSeq ID were used, which are from the Xenogene track from the rheMac10 genome annotation of the University of California, Santa Cruz (UCSC, Santa Cruz, CA, USA).

### Statistical analysis

Statistical analysis was performed using the JMP 16 software (SAS Institute Inc., Cary, NC, USA). Two averages were evaluated by the student’s *t*-test. The averages in various conditions were evaluated by one-way analysis of variance. The correlation was evaluated by R^2^ and p-value for coefficient of correlation. A *p*-value of <0.01 or <0.05 was deemed significant. The standard deviation was shown as error bars in all figures.

### Reporting summary

Further information on research design is available in the Nature Portfolio Reporting Summary linked to this article.

### Supplementary information

Supplementary Information

Reporting Summary

### Source data

Source Data

## Data Availability

Source data are provided with this paper.

## References

[1] Eichler F (2017). Hematopoietic stem-cell gene therapy for cerebral adrenoleukodystrophy. N. Engl. J. Med..

[2] Kanter J (2022). Biologic and clinical efficacy of lentiglobin for sickle cell disease. N. Engl. J. Med..

[3] Kohn DB (2020). Lentiviral gene therapy for X-linked chronic granulomatous disease. Nat. Med..

[4] Kohn DB (2021). Autologous ex vivo lentiviral gene therapy for adenosine deaminase deficiency. N. Engl. J. Med..

[5] Steinberg MH (1999). Management of sickle cell disease. N. Engl. J. Med..

[6] Charache S (1995). Effect of hydroxyurea on the frequency of painful crises in sickle cell anemia. Investigators of the Multicenter Study of Hydroxyurea in Sickle Cell Anemia. N. Engl. J. Med..

[7] Hsieh MM (2014). Nonmyeloablative HLA-matched sibling allogeneic hematopoietic stem cell transplantation for severe sickle cell phenotype. JAMA.

[8] Hsieh MM (2009). Allogeneic hematopoietic stem-cell transplantation for sickle cell disease. N. Engl. J. Med..

[9] Goyal S (2022). Acute myeloid leukemia case after gene therapy for sickle cell disease. N. Engl. J. Med..

[10] Hsieh MM (2020). Myelodysplastic syndrome unrelated to lentiviral vector in a patient treated with gene therapy for sickle cell disease. Blood Adv..

[11] Uchida N (2021). Preclinical evaluation for engraftment of CD34(+) cells gene-edited at the sickle cell disease locus in xenograft mouse and non-human primate models. Cell Rep. Med..

[12] Frangoul H (2021). CRISPR-Cas9 gene editing for sickle cell disease and β-Thalassemia. N. Engl. J. Med..

[13] Copelan EA (2006). Hematopoietic stem-cell transplantation. N. Engl. J. Med..

[14] Boulad F (2022). Lentiviral globin gene therapy with reduced-intensity conditioning in adults with β-thalassemia: a phase 1 trial. Nat. Med..

[15] Drysdale CM, Tisdale JF, Uchida N (2020). Immunoresponse to gene-modified hematopoietic stem cells. Mol. Ther. Methods Clin. Dev..

[16] Uchida N (2019). Busulfan combined with immunosuppression allows efficient engraftment of gene-modified cells in a rhesus macaque model. Mol. Ther..

[17] Uchida N (2016). Total body irradiation must be delivered at high dose for efficient engraftment and tolerance in a rhesus stem cell gene therapy model. Mol. Ther. Methods Clin. Dev..

[18] Ghannam JY (2020). Baseline TP53 mutations in adults with SCD developing myeloid malignancy following hematopoietic cell transplantation. Blood.

[19] Czechowicz A (2019). Selective hematopoietic stem cell ablation using CD117-antibody-drug-conjugates enables safe and effective transplantation with immunity preservation. Nat. Commun..

[20] Uchida, N. et al. Sustained fetal hemoglobin induction in vivo is achieved by *BCL11A* interference and coexpressed truncated erythropoietin receptor. *Sci. Transl. Med.* **13**, eabb0411 (2021). 10.1126/scitranslmed.abb0411 33910976

[21] Saha A (2022). A CD45-targeted antibody-drug conjugate successfully conditions for allogeneic hematopoietic stem cell transplantation in mice. Blood.

[22] Uchida N (2012). High-efficiency transduction of rhesus hematopoietic repopulating cells by a modified HIV1-based lentiviral vector. Mol. Ther..

[23] Uchida N (2009). Development of a human immunodeficiency virus type 1-based lentiviral vector that allows efficient transduction of both human and rhesus blood cells. J. Virol..

[24] Palchaudhuri R (2016). Non-genotoxic conditioning for hematopoietic stem cell transplantation using a hematopoietic-cell-specific internalizing immunotoxin. Nat. Biotechnol..

[25] Uchida N (2013). Integration-specific In vitro evaluation of lentivirally transduced rhesus CD34(+) cells correlates with in vivo vector copy number. Mol. Ther. Nucleic Acids.

[26] Li Y (2019). Myeloid neoplasms in the setting of sickle cell disease: an intrinsic association with the underlying condition rather than a coincidence; report of 4 cases and review of the literature. Mod. Pathol..

[27] Brunson A (2017). Increased risk of leukemia among sickle cell disease patients in California. Blood.

[28] Miettinen M, Lasota J (2005). KIT (CD117): a review on expression in normal and neoplastic tissues, and mutations and their clinicopathologic correlation. Appl. Immunohistochem. Mol. Morphol..

[29] Döhner H (2017). Diagnosis and management of AML in adults: 2017 ELN recommendations from an international expert panel. Blood.

[30] L’Italien L (2018). Mechanistic insights of an immunological adverse event induced by an anti-KIT antibody drug conjugate and mitigation strategies. Clin. Cancer Res..

[31] Sanders JE (1996). Pregnancies following high-dose cyclophosphamide with or without high-dose busulfan or total-body irradiation and bone marrow transplantation. Blood.

[32] Qiao SW (2008). Dependence of antibody-mediated presentation of antigen on FcRn. Proc. Natl Acad. Sci. USA.

[33] Jeffrey SC (2013). A potent anti-CD70 antibody-drug conjugate combining a dimeric pyrrolobenzodiazepine drug with site-specific conjugation technology. Bioconjug Chem..

[34] Tiberghien AC (2016). Design and synthesis of tesirine, a clinical antibody-drug conjugate pyrrolobenzodiazepine dimer payload. ACS Med. Chem. Lett..

[35] Uchida N (2019). Development of a forward-oriented therapeutic lentiviral vector for hemoglobin disorders. Nat. Commun..

[36] Uchida N, Washington KN, Lap CJ, Hsieh MM, Tisdale JF (2011). Chicken HS4 insulators have minimal barrier function among progeny of human hematopoietic cells transduced with an HIV1-based lentiviral vector. Mol. Ther..

[37] Ageyama N (2002). Safe and efficient methods of autologous hematopoietic stem cell transplantation for biomedical research in cynomolgus monkeys. Comp. Med..

[38] Haro-Mora JJ (2020). Biallelic correction of sickle cell disease-derived induced pluripotent stem cells (iPSCs) confirmed at the protein level through serum-free iPS-sac/erythroid differentiation. Stem Cells Transl. Med..

[39] Uchida N (2017). Efficient generation of β-globin-expressing erythroid cells using stromal cell-derived induced pluripotent stem cells from patients with sickle cell disease. Stem Cells.

[40] Voutetakis A (2010). AAV5-mediated gene transfer to the parotid glands of non-human primates. Gene Ther..

[41] De Ravin SS (2016). Lentiviral hematopoietic stem cell gene therapy for X-linked severe combined immunodeficiency. Sci. Transl. Med..

