# Supplementary material for: Fertility-preserving myeloablative conditioning using single-dose CD117 antibody-drug conjugate in a rhesus gene therapy model
Source: Nat Commun. 2023 Oct 12;14:6291. doi: 10.1038/s41467-023-41153-5 (PMC10570335; doi:10.1038/s41467-023-41153-5)
Supplement: Supplementary file 1 — Supplementary Information [file 41467_2023_41153_MOESM1_ESM.pdf]

Supplementary information

Fertility-preserving myeloablative conditioning using single-dose CD117 antibody-drug conjugate in a rhesus gene therapy model

Naoya Uchida[1,2]*, Ulana Stasula[1], Selami Demirci[1], Paula Germino-Watnick[1], Malikiya Hinds[1], Anh Le[1], Rebecca Chu[1], Alexander Berg[1], Xiong Liu[1], Ling Su[3], Xiaolin Wu[3], Allen E Krouse[4], N Seth Linde[4], Aylin Bonifacino[4], So Gun Hong[4], Cynthia E Dunbar[4], Leanne Lanieri[5], Anjali Bhat[5], Rahul Palchaudhuri[5], Bindu Bennet[5], Megan Hoban[5], Kirk Bertelsen[5], Lisa M Olson[5], Robert E Donahue[1], John F Tisdale[1]

1) Cellular and Molecular Therapeutics Branch, NHLBI/NIDDK, NIH, Bethesda, MD, USA
2) Division of Molecular and Medical Genetics, Center for Gene and Cell Therapy, The Institute of Medical Science, The University of Tokyo, Tokyo, Japan
3) Genomics Technology Laboratory, Leidos Biomedical Research, Inc., Frederick National Laboratory for Cancer Research, Frederick, MD, USA
4) Translational Stem Cell Biology Branch, NHLBI, NIH, Bethesda, MD, USA
5) Magenta Therapeutics, Cambridge, Massachusetts (MA), USA

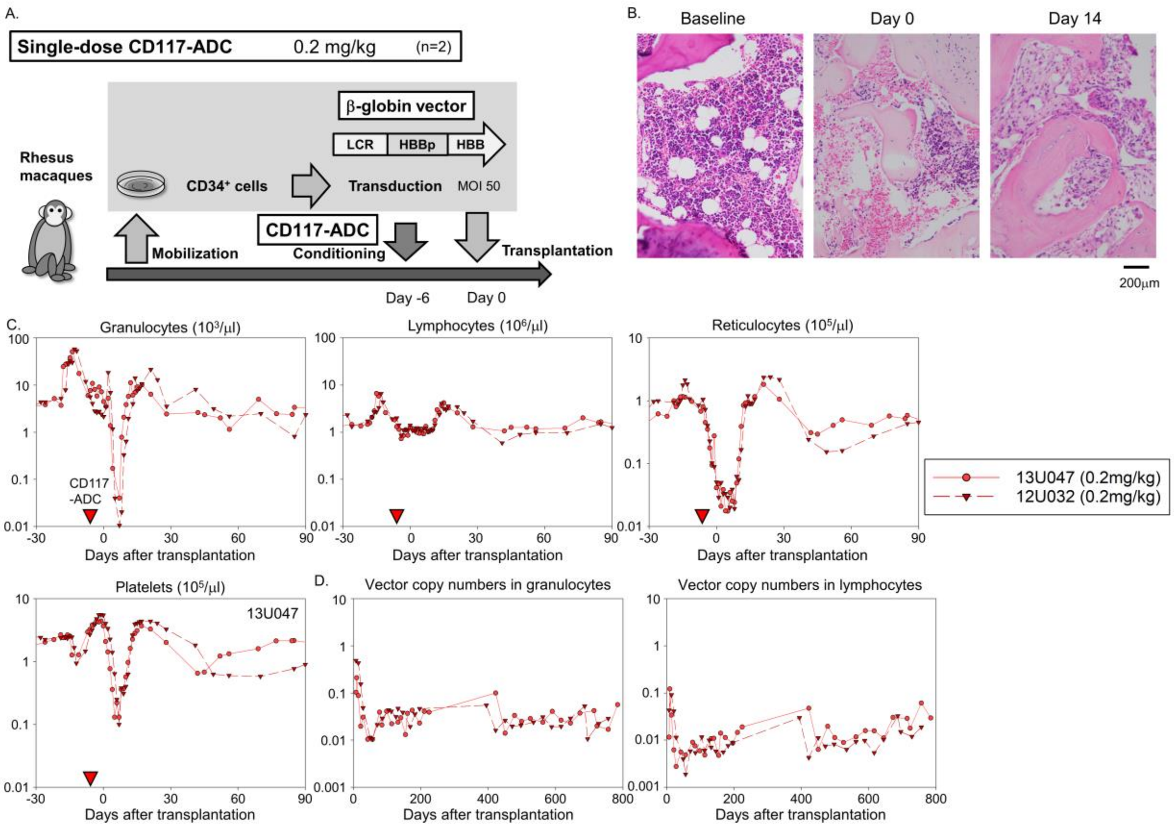

Supplementary Figure 1. Impact of 0.2 mg/kg CD117-ADC conditioning on rhesus macaque marrow hematopoietic stem/progenitor cell content and engraftment of lentivirally-transduced autologous CD34+ cells. (A) Experimental design. Rhesus macaques (n=2), 13U047 and 12U032, were mobilized and CD34+ cells (3.4±0.7e6/kg) collected by apheresis from the peripheral blood were purified and transduced with a lentiviral vector encoding a human β-globin gene (*HBB*) at MOI 50. Transduced CD34+ cells (VCN 4.7±1.2) were transplanted into autologous macaques 6 days after a single intravenous dose of 0.2 mg/kg CD117-ADC. (B) Hematoxylin and eosin-stained bone marrow biopsies sampled at baseline, transplant day (day 0), and day 14 post-transplant (12U032), and shown at 20x magnification (similar observation at n=2 biologically independent animals). (C) Peripheral blood counts of granulocytes, lymphocytes, reticulocytes, and platelets in rhesus macaques beginning before CD117-ADC (day -6) and through transplantation and recovery. (D) Gene marking levels (VCN) in granulocytes and lymphocytes post-transplantation, evaluated by qPCR. LCR: locus control regions, HBBp: the *HBB* promoter. Source data are provided as a Source Data file.

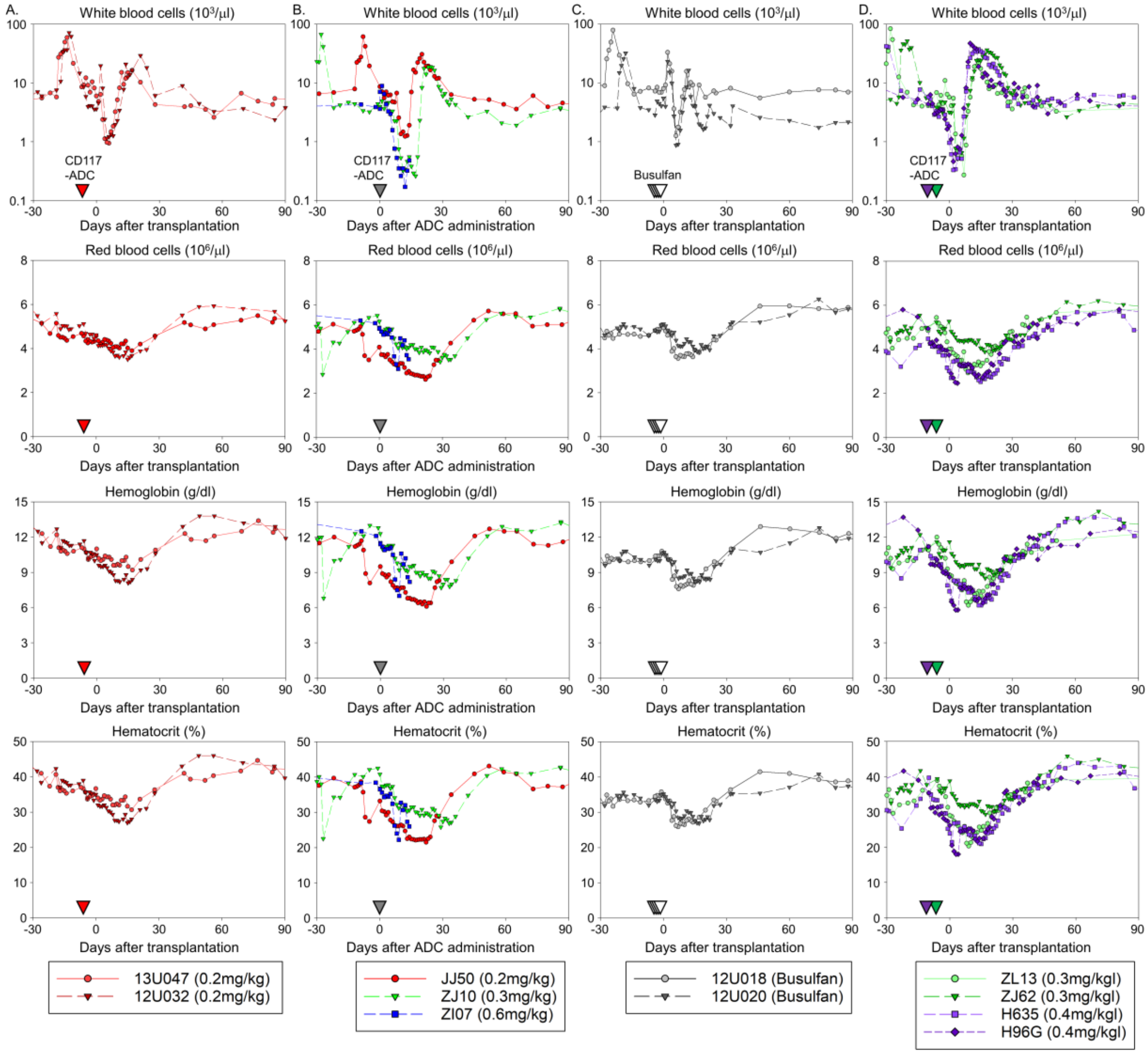

Supplementary Figure 2. Additional blood counts in rhesus macaques following conditioning. (A-D) Blood counts (white blood cells, red blood cells, hemoglobin concentrations, and hematocrit) before and after transplantation with (A) 0.2 mg/kg CD117-ADC (13U047 and 12U032), (C) myeloablative busulfan 5.5 mg/kg x 4 days (12U018 and 12U020), and (D) 0.3-0.4 mg/kg CD117-ADC (0.3 mg/kg in ZL13 and ZJ62, 0.4 mg/kg in H635 and H96G), as well as (B) before and after CD117-ADC administration (0.2 mg/kg in JJ50, 0.3 mg/kg in ZJ10, and 0.6 mg/kg in ZI07) without autologous CD34+ cell infusion. Source data are provided as a Source Data file.

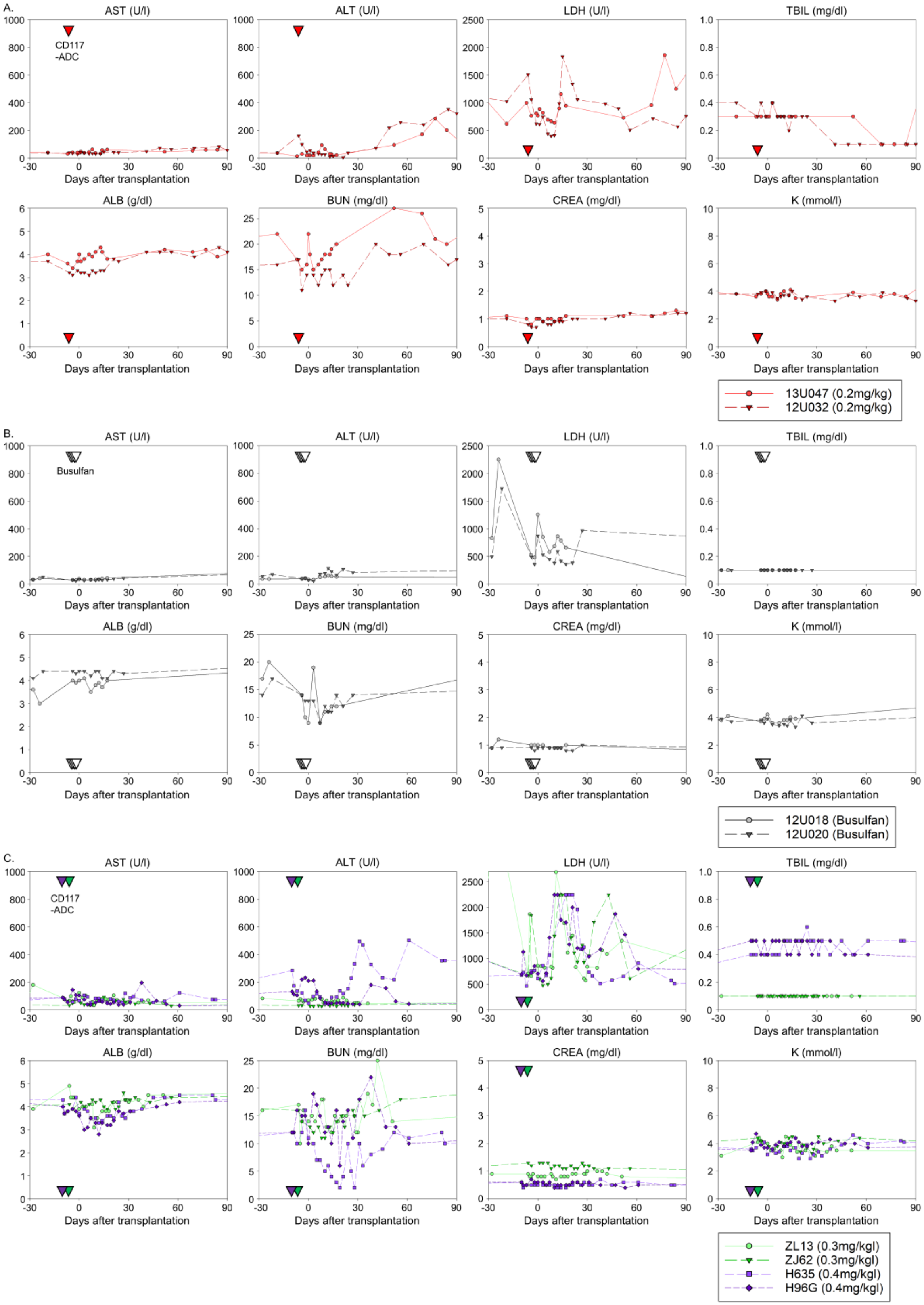
A.
AST (U/l)
CD117
-ADC
ALT (U/l)
LDH (U/l)
TBIL (mg/dl)
ALB (g/dl)
BUN (mg/dl)
CREA (mg/dl)
K (mmol/l)
Days after transplantation
13U047 (0.2mg/kg)
12U032 (0.2mg/kg)
B.
AST (U/l)
Busulfan
ALT (U/l)
LDH (U/l)
TBIL (mg/dl)
ALB (g/dl)
BUN (mg/dl)
CREA (mg/dl)
K (mmol/l)
Days after transplantation
12U018 (Busulfan)
12U020 (Busulfan)
C.
AST (U/l)
CD117
-ADC
ALT (U/l)
LDH (U/l)
TBIL (mg/dl)
ALB (g/dl)
BUN (mg/dl)
CREA (mg/dl)
K (mmol/l)
Days after transplantation
ZL13 (0.3mg/kgl)
ZJ62 (0.3mg/kgl)
H635 (0.4mg/kgl)
H96G (0.4mg/kgl)

Supplementary Figure 3. Liver and kidney tests before and after CD34+ cell transplantation in rhesus macaques. (A-C) Liver enzymes (AST, ALT, and LDH), liver function tests (TBIL and ALB), and kidney function tests (BUN, CREA, and K) before and after transplantation with (A) 0.2 mg/kg CD117-ADC (13U047 and 12U032), (B) myeloablative busulfan (12U018 and 12U020), and (C) 0.3-0.4 mg/kg CD117-ADC (0.3 mg/kg in ZL13 and ZJ62, 0.4 mg/kg in H635 and H96G). Source data are provided as a Source Data file.

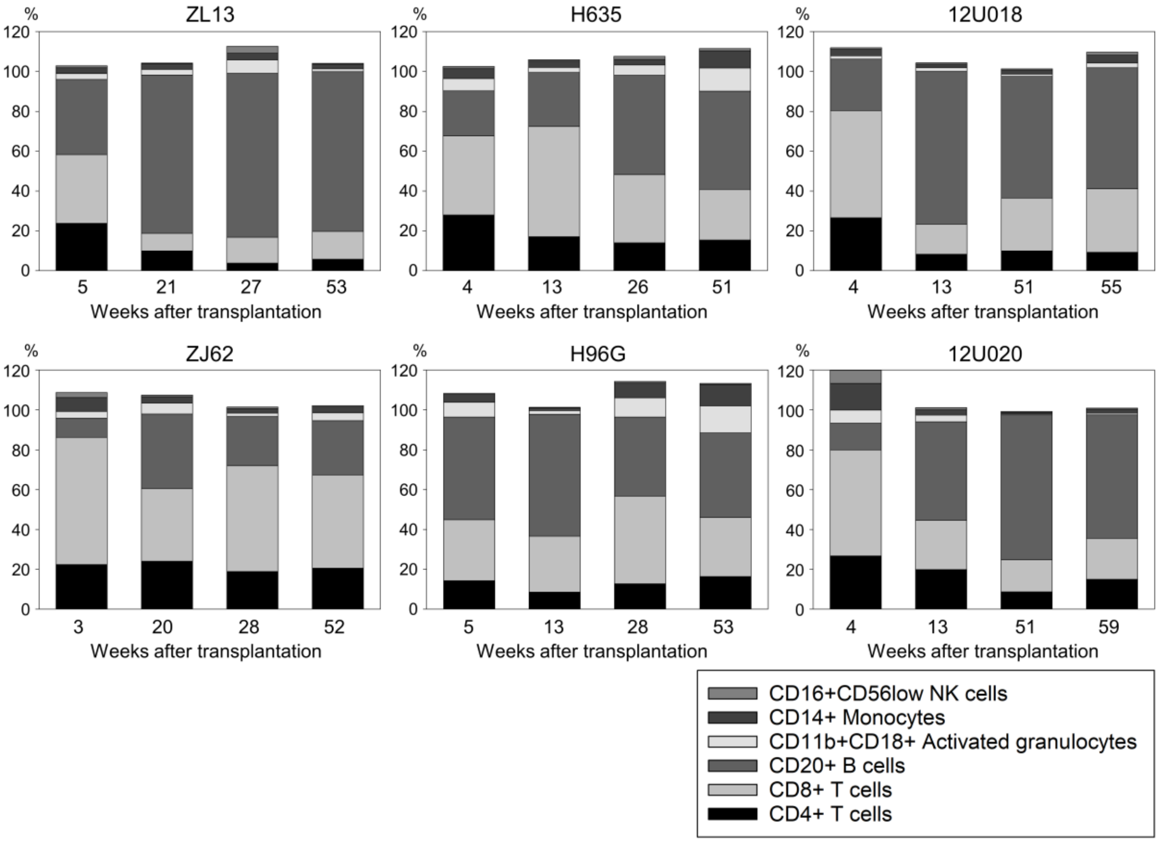

Supplementary Figure 4. Subset analysis of peripheral blood mononuclear cells (PBMCs) in transplanted animals. Subset analysis (CD4+ T cells, CD8+ T cells, CD20+ B cells, CD11b+CD18+ activated granulocytes, CD14+ monocytes, and CD16+CD56low natural killer (NK) cells) of PBMCs at various time points after transplantation with CD117-ADC (0.3 mg/kg in ZL13 and ZJ62, 0.4 mg/kg in H635 and H96G) and myeloablative busulfan (12U018 and 12U020). Source data are provided as a Source Data file.

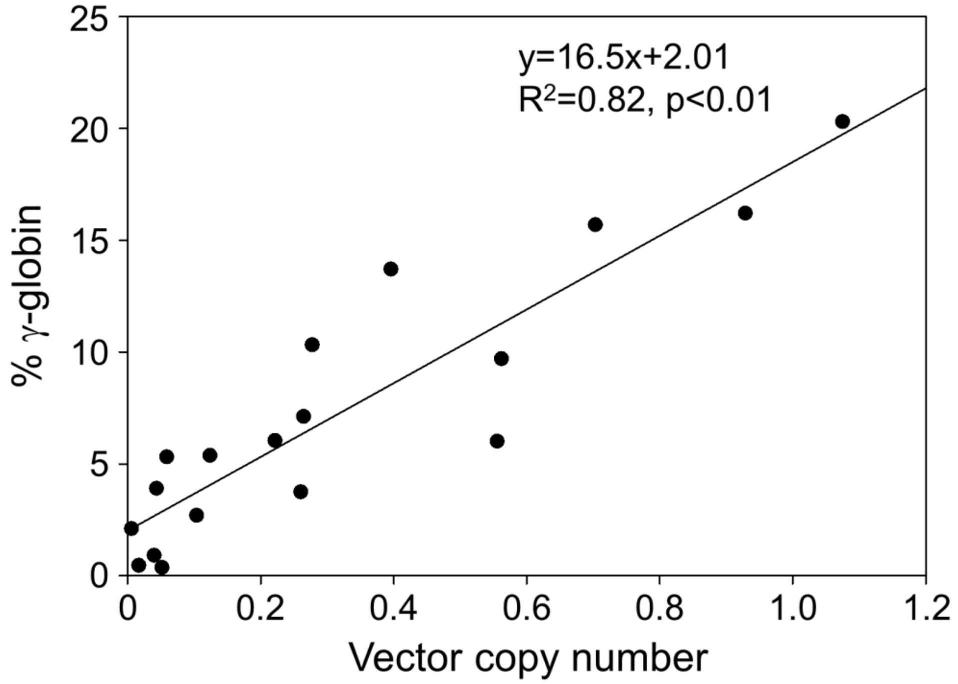

Supplementary Figure 5. Positive correlation between HbF induction levels and granulocyte VCN in rhesus macaques. The relationship between HPLC-measured HbF (γ-globin) amounts and granulocyte VCN of the thEpoR/shmiR-BCL11A vector, using three timepoints for each transplanted animal (ZL13, ZJ62, H635, H96G, 12U018, and 12U020). The correlation was evaluated by R^2 and p-value for coefficient of correlation. p=2.08e-7. Source data are provided as a Source Data file.

A.

Animal ID	ZL13	ZJ62	H635	H96G	12U018	12U020
VCN	0.017	0.124	0.265	0.060	1.075	0.282
Total IS	405	2938	4765	872	6368	4398
Unique IS	221	1073	3553	338	4159	2553
Max IS counts	13	38	23	32	19	22
Max IS%	3.21%	1.29%	0.48%	3.67%	0.30%	0.50%
Min IS counts	1	1	1	1	1	1
UC_{50}	56	180	1171	54	1087	604
Simpson's diversity index	0.99247	0.99773	0.99959	0.99200	0.99961	0.99928

B.

IS	ZL13	ZJ62	H635	H96G	12U018	12U020
1	PHIP (3.2%)	DACH1 (1.3%)	CCDC59 (0.5%)	PPP2R2A (3.7%)	CHM (0.3%)	LINC01526 (0.5%)
2	LINC02760 (2.5%)	GPD2 (1.2%)	MARCHF1 (0.3%)	SESTD1 (2.6%)	COBLL1 (0.3%)	SERPINI1 (0.5%)
3	CPNE4 (2.2%)	NDUFB4 (0.9%)	TRPM6 (0.3%)	MIR31HG (2.2%)	LOC105370457 (0.3%)	KANSL1L (0.4%)
4	COL8A1 (1.5%)	FARS2 (0.9%)	ZNF654 (0.2%)	NDST3 (1.9%)	RIT2 (0.3%)	RPL13AP20 (0.4%)
5	LOC100130111(1.5%)	RBMS1 (0.8%)	THSD7B (0.2%)	GLRB (1.9%)	PIK3CA (0.2%)	C15orf41 (0.4%)
6	PTGS2 (1.2%)	PDGFC (0.7%)	SYNPO2 (0.2%)	TNIP3 (1.8%)	MTHFD2L (0.2%)	MCM8 (0.3%)
7	LRP1B (1.2%)	INTU (0.7%)	MRPL19 (0.2%)	HAS2 (1.7%)	PCDH11Y (0.2%)	PTPRQ (0.3%)
8	MTMR2 (1.2%)	INTU (0.7%)	LINC02476 (0.2%)	MPEG1 (1.6%)	NABP1 (0.2%)	ACADL (0.3%)
9	XIST (1.2%)	KANSL1L(0.6%)	ABR (0.1%)	ZNF277 (1.5%)	CDH9 (0.2%)	ZBTB20 (0.3%)
10	LINC02760 (1.0%)	PCDH9 (0.6%)	MIB1 (0.1%)	DDX3X (1.5%)	LINC01478 (0.1%)	DPYD (0.3%)

Supplementary Figure 6. High diversity of lentiviral integration sites (IS) in transplanted animals. (A) IS analysis of lentiviral vectors in granulocytes one year after transplantation with CD117-ADC (0.3 mg/kg in ZL13 and ZJ62, 0.4 mg/kg in H635 and H96G) and myeloablative busulfan (12U018 and 12U020). Diversity was evaluated by Simpson's diversity index (0 representing no diversity, and 1 representing infinite diversity). (B) Gene names of top 10 ISs along with IS percentages. VCN: average vector copy number per cell, UC_{50}: unique clone number of top 50%. Source data are provided as a Source Data file.

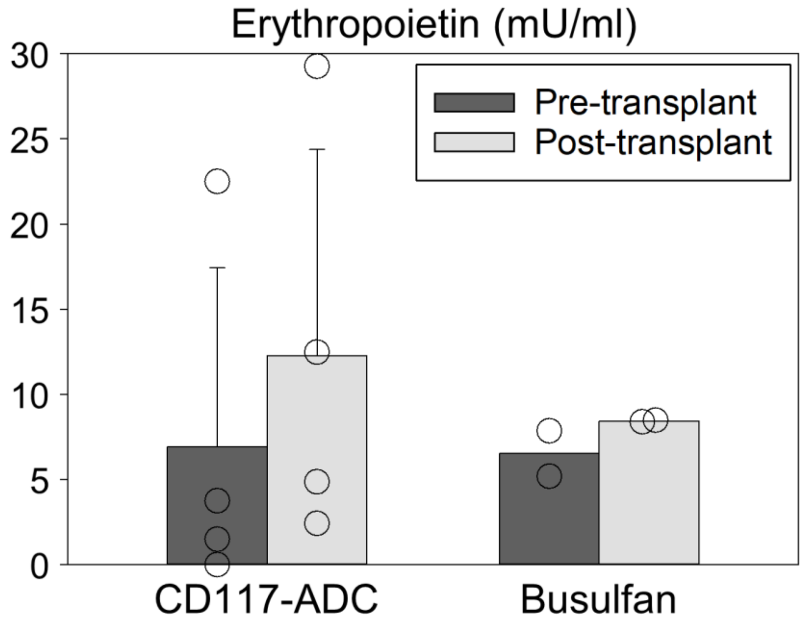

Supplementary Figure 7. Similar erythropoietin levels between before and after transplant with thEpoR/shmiR-BCL11A gene addition. Erythropoietin levels in rhesus serum pre-transplant and 14-22 months post-transplant with thEpoR/shmiR-BCL11A gene addition following CD117-ADC conditioning (0.3 mg/kg in ZL13 and ZJ62, 0.4 mg/kg in H635 and H96G, n=4 biologically independent animals) and myeloablative busulfan conditioning (12U018 and 12U020, n=2 biologically independent animals). Data are presented as mean +/- standard deviation. Not significant (n.s.) evaluated by one-tailed paired *t*-test between pre- and post-transplant for CD117-ADC animals. Source data are provided as a Source Data file.

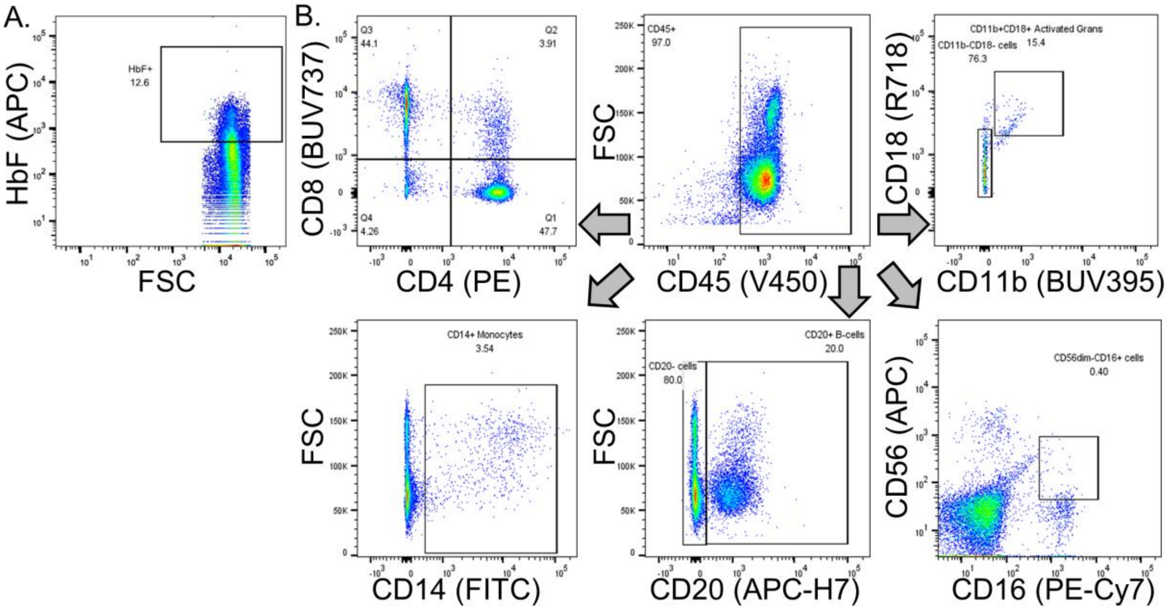

Supplementary Figure 8. Gating strategies in flow cytometry. (A) A flow cytometry panel for HbF analysis. (B) Flow cytometry panels for PBMC subset analysis, including CD4+ T cells, CD8+ T cells, CD20+ B cells, CD11b+CD18+ activated granulocytes, CD14+ monocytes, and CD16+CD56low NK cells. APC: allophycocyanin, FSC: forward scatter, PE: phycoerythrin, V450: Violet 450, R718: Red 718, FITC: fluorescein isothiocyanate, PE-Cy7: PE-Cyanine 7.
