# Supplementary material for: Fertility-preserving myeloablative conditioning using single-dose CD117 antibody-drug conjugate in a rhesus gene therapy model
Source: Nat Commun. 2023 Oct 12;14:6291. doi: 10.1038/s41467-023-41153-5 (PMC10570335; doi:10.1038/s41467-023-41153-5)
Supplement: Supplementary file 2 — Reporting Summary [file 41467_2023_41153_MOESM2_ESM.pdf]

nature portfolio

Corresponding author(s): Naoya Uchida

Last updated by author(s): Jul 27, 2023

Reporting Summary

Nature Portfolio wishes to improve the reproducibility of the work that we publish. This form provides structure for consistency and transparency in reporting. For further information on Nature Portfolio policies, see our Editorial Policies and the Editorial Policy Checklist.

Statistics

For all statistical analyses, confirm that the following items are present in the figure legend, table legend, main text, or Methods section.

n/a	Confirmed	
☐	☒	The exact sample size (*n*) for each experimental group/condition, given as a discrete number and unit of measurement
☐	☒	A statement on whether measurements were taken from distinct samples or whether the same sample was measured repeatedly
☐	☒	The statistical test(s) used AND whether they are one- or two-sided<br>*Only common tests should be described solely by name; describe more complex techniques in the Methods section.*
☒	☐	A description of all covariates tested
☐	☒	A description of any assumptions or corrections, such as tests of normality and adjustment for multiple comparisons
☐	☒	A full description of the statistical parameters including central tendency (e.g. means) or other basic estimates (e.g. regression coefficient) AND variation (e.g. standard deviation) or associated estimates of uncertainty (e.g. confidence intervals)
☐	☒	For null hypothesis testing, the test statistic (e.g. F, t, r) with confidence intervals, effect sizes, degrees of freedom and P value noted<br>*Give P values as exact values whenever suitable.*
☒	☐	For Bayesian analysis, information on the choice of priors and Markov chain Monte Carlo settings
☒	☐	For hierarchical and complex designs, identification of the appropriate level for tests and full reporting of outcomes
☒	☐	Estimates of effect sizes (e.g. Cohen's d, Pearson's r), indicating how they were calculated

Our web collection on statistics for biologists contains articles on many of the points above.

Software and code

Policy information about availability of computer code

Data collection	No software was used.
Data analysis	Statistical analysis was performed using the JMP 16 software (SAS Institute Inc.). Flow cytometry data were analyzed by FlowJo v10 (BD Biosciences). The EC50 and EC90 values were calculated with GraphPad Prism v8 (GraphPad Software).

For manuscripts utilizing custom algorithms or software that are central to the research but not yet described in published literature, software must be made available to editors and reviewers. We strongly encourage code deposition in a community repository (e.g. GitHub). See the Nature Portfolio guidelines for submitting code & software for further information.

Data

Policy information about availability of data

All manuscripts must include a data availability statement. This statement should provide the following information, where applicable:

- Accession codes, unique identifiers, or web links for publicly available datasets
- A description of any restrictions on data availability
- For clinical datasets or third party data, please ensure that the statement adheres to our policy

Source data are provided with this paper. The rhesus genome assembly rheMac10 is available in National Center for Biotechnology Information Datasets (https://www.ncbi.nlm.nih.gov/datasets/genome/GCF_003339765.1/).

Human research participants

Policy information about studies involving human research participants and Sex and Gender in Research.

Reporting on sex and gender	We didn't report the gender in human CD34+ cells, because these cells were used to evaluate the cross reactivity of CD117-ADC with rhesus CD34+ cells.
Population characteristics	Human CD34+ cells were originated from steady-state bone marrow in healthy donors.
Recruitment	It is based on STEMCELL technologies where we purchased human CD34+ cells.
Ethics oversight	It is based on STEMCELL technologies where we purchased human CD34+ cells.

Note that full information on the approval of the study protocol must also be provided in the manuscript.

Field-specific reporting

Please select the one below that is the best fit for your research. If you are not sure, read the appropriate sections before making your selection.

☒ Life sciences ☐ Behavioural & social sciences ☐ Ecological, evolutionary & environmental sciences

For a reference copy of the document with all sections, see nature.com/documents/nr-reporting-summary-flat.pdf

Life sciences study design

All studies must disclose on these points even when the disclosure is negative.

Sample size	We used 3 human donors and 2 rhesus donors for the CD34+ cell killing assay in vitro, 3 cynomolgus macaques for each group in CD34+CD90+CD45RA- cell depletion assay in vivo, 3 cynomolgus macaques for each group in dose-escalating toxicity assay, 1 rhesus macaque for each group in clearance assay, 1 rhesus macaque for each group in dose-escalating toxicity assay, and 2 rhesus macaques for each group in CD34+ cells transplantation with lentiviral gene marking.<br><br>Human CD34+ cells are limited resources due to the requirement of a healthy donor. In addition, non-human primates are limited, because they were preferentially used for COVID-19 vaccination studies. Therefore, we used minimal but sufficient numbers of donors (2-3 donors) for in vitro CD34+ cells experiments, as well as animals (1-3) for in vivo experiments.
Data exclusions	No data was excluded from the analysis.
Replication	We reproducibly observed the engraftment of lentivirally gene-modified cells in transplanted rhesus macaques following CD117-ADC conditioning (6 replicates).
Randomization	We evaluated multiple doses of CD117-ADC in identical conditions for each experiment. Randomization is not relevant to this non-clinical study.
Blinding	Blinding is not relevant to this non-clinical study, since there was no allocation of participants to experimental groups. Therefore, we carefully designed each experiment to collect objective data, such as cell viability by flow cytometry, vector copy number by quantitative PCR, and globin protein amounts by HPLC. In fact, technicians for data collection and analysis were mostly blinded, since only limited information (animal ID and analysis methods) was provided. However, there is no formal blinding.

Reporting for specific materials, systems and methods

We require information from authors about some types of materials, experimental systems and methods used in many studies. Here, indicate whether each material, system or method listed is relevant to your study. If you are not sure if a list item applies to your research, read the appropriate section before selecting a response.

Materials & experimental systems

n/a	Involved in the study	
☐	☒	Antibodies
☐	☒	Eukaryotic cell lines
☒	☐	Palaeontology and archaeology
☐	☒	Animals and other organisms
☒	☐	Clinical data
☒	☐	Dual use research of concern

Methods

n/a	Involved in the study	
☒	☐	ChIP-seq
☐	☒	Flow cytometry
☒	☐	MRI-based neuroimaging

Antibodies

Antibodies used

We used antibodies specific for CD34 (clone 561, Biolegend, 343626), CD90 (Clone 5E10, BioLegend, 328114), CD45RA (clone T6D11, Miltenyi Biotec, 130-113-360), fetal hemoglobin (clone 2D12, BD Biosciences, 551796), CD4 (clone L200, BD Biosciences, 550630), CD8 (clone G42-8, BD Biosciences, 749034), CD11b (clone D12, BD Biosciences, 742643), CD14 (clone M5E2, BD Biosciences, 561712), CD16 (clone 3G8, BD Biosciences, 560918) CD18 (clone 6.7, BD Biosciences, 752220), CD20 (clone 2H7, BD Biosciences, 560734/560853), CD45 (clone D058-1283, BD Biosciences, 561291), and CD56 (clone B159, BD Biosciences, 555518).

Validation

The antibodies are validated on the manufacturer's website.

CD34, Reactivity: Human, Cynomolgus, and Rhesus, Application: flow cytometry, Full description: https://www.biolegend.com/fr-ch/products/brilliant-violet-785-anti-human-cd34-antibody-13790

CD90, Reactivity: Human, African Green, Baboon, Cynomolgus, Pigtailed Macaque, Rhesus, and Pig, Application: flow cytometry, Full description: https://www.biolegend.com/en-us/products/apc-anti-human-cd90-thy1-antibody-4116

CD45RA, Reactivity: Human, Cynomolgus, and Rhesus, Application: flow cytometry, Full description: https://www.miltenyibiotec.com/US-en/products/cd45ra-antibody-anti-human-t6d11.html#conjugate=vioblue:size=100-tests-in-200-ul

Fetal hemoglobin, Reactivity: Human, Application: Flow cytometry, Full description: https://www.bdbiosciences.com/en-us/products/reagents/flow-cytometry-reagents/research-reagents/single-color-antibodies-ruo/purified-mouse-anti-human-fetal-hemoglobin.551796

CD4, Reactivity: Human, Rhesus, Cynomolgus, and Baboon, Application: Flow cytometry, Full description: https://www.bdbiosciences.com/en-us/products/reagents/flow-cytometry-reagents/research-reagents/single-color-antibodies-ruo/pe-mouse-anti-human-cd4.550630

CD8, Reactivity: Human, Application: Flow cytometry, Full description: https://www.bdbiosciences.com/en-us/products/reagents/flow-cytometry-reagents/research-reagents/single-color-antibodies-ruo/buv737-mouse-anti-human-cd8.749034

CD11b, Reactivity: Human, Application: Flow cytometry, Full description: https://www.bdbiosciences.com/en-us/products/reagents/flow-cytometry-reagents/research-reagents/single-color-antibodies-ruo/buv395-mouse-anti-human-cd11b.742643

CD14, Reactivity: Human, Rhesus, Cynomolgus, Baboon, and Dog, Application: Flow cytometry, Full description: https://www.bdbiosciences.com/en-us/products/reagents/flow-cytometry-reagents/research-reagents/single-color-antibodies-ruo/fitc-mouse-anti-human-cd14.561712

CD16, Reactivity: Human, Application: Flow cytometry, Full description: https://www.bdbiosciences.com/en-us/products/reagents/flow-cytometry-reagents/research-reagents/single-color-antibodies-ruo/pe-cy-7-mouse-anti-human-cd16.560918

CD18, Reactivity: Human, Application: Flow cytometry, Full description: https://www.bdbiosciences.com/en-us/products/reagents/flow-cytometry-reagents/research-reagents/single-color-antibodies-ruo/r718-mouse-anti-human-cd18.752220

CD20, Reactivity: Human, Rhesus, Cynomolgus, and Baboon, Application: Flow cytometry, Full description: https://www.bdbiosciences.com/en-us/products/reagents/flow-cytometry-reagents/research-reagents/single-color-antibodies-ruo/apc-h7-mouse-anti-human-cd20.560734

CD45, Reactivity: Rhesus, Cynomolgus, and Baboon, Application: Flow cytometry, Full description: https://www.bdbiosciences.com/en-us/products/reagents/flow-cytometry-reagents/research-reagents/single-color-antibodies-ruo/v450-mouse-anti-nhp-cd45.561291

CD56, Reactivity: Human, Application: Flow cytometry, Full description: https://www.bdbiosciences.com/en-us/products/reagents/flow-cytometry-reagents/research-reagents/single-color-antibodies-ruo/apc-mouse-anti-human-cd56-ncam-1.555518

Eukaryotic cell lines

Policy information about cell lines and Sex and Gender in Research

Cell line source(s)

We obtained 293T and HeLa cell lines from American Type Culture Collection (ATCC).

Authentication	Both 293T and HeLa cell lines were authenticated by morphology.
Mycoplasma contamination	No mycoplasma contamination was confirmed by ATCC and our laboratory.
Commonly misidentified lines (See ICLAC register)	We didn't use any commonly misidentified cell lines in this study.

Animals and other research organisms

Policy information about studies involving animals; ARRIVE guidelines recommended for reporting animal research, and Sex and Gender in Research

Laboratory animals	We used 18 male cynomolgus macaques for CD34+CD90+CD45RA- cell depletion assay and dose-escalating toxicity assay that were 2.5-4 years old. We also used 2 male and 6 female rhesus macaques for transplantation that were 3-8 years old.
Wild animals	The study did not involve wild animals.
Reporting on sex	The use of female monkeys allows us to evaluate menstrual cycles following transplantation.
Field-collected samples	The study did not involve samples collected from the field.
Ethics oversight	We follow the guidelines set out by the Public Health Service Policy on Humane Care and Use of Laboratory Animals under a protocol (H-0136) approved by the Animal Care and Use Committee of National Heart, Lung, and Blood Institute (NHLBI).

Note that full information on the approval of the study protocol must also be provided in the manuscript.

Flow Cytometry

Plots

Confirm that:

☒ The axis labels state the marker and fluorochrome used (e.g. CD4-FITC).

☒ The axis scales are clearly visible. Include numbers along axes only for bottom left plot of group (a 'group' is an analysis of identical markers).

☒ All plots are contour plots with outliers or pseudocolor plots.

☒ A numerical value for number of cells or percentage (with statistics) is provided.

Methodology

Sample preparation	The cells were incubated with fluorescent-conjugated antibodies according to the company protocol.
Instrument	We used FACSCelesta (BD Biosciences) in CD34+ cell killing assay and FACSCanto (BD Biosciences) in rhesus transplantation.
Software	The FlowJo v10 (BD Biosciences) was used to analyze flow cytometry data.
Cell population abundance	We analyzed 1e4 cells in flow cytometry.
Gating strategy	Unstained cells were used as a gating control.

☒ Tick this box to confirm that a figure exemplifying the gating strategy is provided in the Supplementary Information.
